# Genomic analyses of agronomic traits in tea plants and related *Camellia* species

**DOI:** 10.3389/fpls.2024.1449006

**Published:** 2024-08-26

**Authors:** Shengchang Duan, Liang Yan, Zongfang Shen, Xuzhen Li, Baozheng Chen, Dawei Li, Hantao Qin, Muditha K. Meegahakumbura, Moses C. Wambulwa, Lianming Gao, Wei Chen, Yang Dong, Jun Sheng

**Affiliations:** ^1^ College of Plant Protection, Yunnan Agricultural University, Kunming, China; ^2^ State Key Laboratory for Conservation and Utilization of Bio-Resources in Yunnan, Yunnan Agricultural University, Kunming, China; ^3^ Yunnan Research Institute for Local Plateau Agriculture and Industry, Kunming, China; ^4^ College of Tea (Pu’er), West Yunnan University of Applied Sciences, Pu’er, China; ^5^ Pu’er Institute of Pu-erh Tea, Pu’er, China; ^6^ CAS Key Laboratory for Plant Diversity and Biogeography of East Asia, Kunming Institute of Botany, Chinese Academy of Science, Kunming, China; ^7^ Germplasm Bank of Wild Species, Kunming Institute of Botany, Chinese Academy of Science, Kunming, China; ^8^ University of Chinese Academy of Science, Beijing, China; ^9^ College of Food Science and Technology, Yunnan Agricultural University, Kunming, China; ^10^ Department of Export Agriculture, Faculty of Animal Science and Export Agriculture, Uva Wellassa University, Badulla, Sri Lanka; ^11^ Department of Life Sciences, School of Science and Computing, South Eastern Kenya University, Kitui, Kenya; ^12^ Lijiang Forest Biodiversity National Observation and Research Station, Kunming Institute of Botany, Chinese Academy of Sciences, Lijiang, China

**Keywords:** *Camellia*, *Camellia sinensis* var. *assamica* cv. ‘Yunkang10’, resequencing, pangenome, assembly

## Abstract

The genus *Camellia* contains three types of domesticates that meet various needs of ancient humans: the ornamental *C. japonica*, the edible oil-producing *C. oleifera*, and the beverage-purposed tea plant *C. sinensis*. The genomic drivers of the functional diversification of *Camellia* domesticates remain unknown. Here, we present the genomic variations of 625 *Camellia* accessions based on a new genome assembly of *C. sinensis* var. *assamica* (‘YK10’), which consists of 15 pseudo-chromosomes with a total length of 3.35 Gb and a contig N50 of 816,948 bp. These accessions were mainly distributed in East Asia, South Asia, Southeast Asia, and Africa. We profiled the population and subpopulation structure in tea tree *Camellia* to find new evidence for the parallel domestication of *C. sinensis* var. *assamica* (CSA) and *C. sinensis* var. *sinensis* (CSS). We also identified candidate genes associated with traits differentiating CSA, CSS, oilseed *Camellia*, and ornamental *Camellia* cultivars. Our results provide a unique global view of the genetic diversification of *Camellia* domesticates and provide valuable resources for ongoing functional and molecular breeding research.

## 1 Introduction

Human ancestors working in agriculture transformed wild plant species into new forms and established all major domesticated crops with desirable traits to meet human needs worldwide (Doebley et al., 2006). Prominent examples include rice, wheat, and maize as staple foods (Doebley et al., 2006), peanut and sesame for oil production (Wei et al., 2015), and cotton for natural fibers (Wang et al., 2019). Through artificial selection, these crops obtain better grain quality (changes in starch and other compounds), higher yield, and altered flowering time or plant height (Gross and Olsen, 2010) due to underlying genetic changes. Crop improvement and diversification would produce different varieties within a single species, but the genetic drivers of trait diversification remain unknown in many less-studied horticultural plants.

The *Camellia* plants in the Theaceae family are native to East Asia, with more than 300 species worldwide (Zeng and Endo, 2019). Three types of plants, including ornamental *Camellia*, oilseed *Camellia*, and tea plants, played indispensable roles in meeting the varied needs of human life (Teixeira and Sousa, 2021). The ornamental *Camellia*, prized for its exquisite, long-lasting flowers and deep green foliage, boasts over 30,000 cultivars in various flower forms and colors. The oilseed *Camellia* (i.e., *C. oleifera*) has been continuously selected for larger seeds with high content and quality of unsaturated fatty acid over the centuries (Lin et al., 2022). The tea plant (*C. sinensis*), including two varieties, *C. sinensis* var. *assamica* (CSA) and *C. sinensis* var. *sinensis* (CSS), has been commercially grown in more than 60 countries as a popular beverage (Drew, 2019). Despite the trait diversification of *Camellia* domesticates, the underlying genomic basis for the evolutionary course remains poorly understood.

Researchers reported many *Camellia* genomes recently from tea plants (Xia et al., 2017; Wei et al., 2018; Wang et al., 2020; Xia et al., 2020; Zhang et al., 2020a, 2020, 2021) and oilseed *Camellia* (Lin et al., 2022). These studies identified genes involved in the biosynthesis of terpenoids and fatty acids, but it is unknown if they bear signatures of human selection. Among tea plants, ‘Yunkang10’ (CSA, ‘YK10’) is a widely cultivated cultivar in Southwestern China. It was recognized as a national fine variety and bred from Fengqing large leaf variety of Yunnan Academy of Agricultural Sciences Tea Research Institute (Zhang et al., 2020c; Lei et al., 2021). It bears the advantages of wide-range adaptability, low-cost cultivation, and quick growth rate over other cultivars (Zhang et al., 2020c). The reported genome of ‘YK10’ (Xia et al., 2017) had poor continuity (contigs N50 = 19,958 bp), and the lack of high-quality CSA genomes warrants obtaining a high-quality genome assembly of the ‘YK10’.

In addition, analyses of population structure and genetic diversity in tea plants yielded two competing theories for its evolution history (Wang et al., 2020; Xia et al., 2020; Zhang et al., 2020b, 2021). The researchers proposed that either two tea varieties (CSS and CSA) underwent parallel domestication (Zhang et al., 2021) or they resulted from a single origin in Southwestern China (Xia et al., 2020). In comparison, oilseed *Camellia* and ornamental *Camellia* populations showed a high genetic diversity (Lin et al., 2022; Yang et al., 2022). Previous reports also showed that simple sequence repeat (SSR) markers could differentiate ornamental *Camellia* populations in northern and southern China according to their geographical origin and genetic background (Yang et al., 2022). In contrast, the *C. oleifera* cultivars were mainly distinguished by their morphological characteristics rather than geographic origin, based on 25,581 SNPs from transcriptome sequencing data of 221 oilseed *Camellia* cultivars (Lin et al., 2022). Despite these advances, additional analyses of a comprehensive collection of cultivars may add to our understanding of the complex evolutionary history of *Camellia*.

Here, we report the genome of ‘YK10’, the resequencing of 625 *Camellia* accessions, and the pan-genome of 206 core cultivars. We evaluated the phylogeny, population structure, and trait diversification of *Camellia* species, which provided insights into the evolution, domestication, and dispersal of this economically significant perennial horticultural plant.

## 2 Materials and methods

### 2.1 DNA isolation, sequencing, and assembly

The leaves of ‘YK10’ were sampled from Lincang, Yunnan province of China. Genomic DNA for sequencing was extracted from leaves using the cetyltrimethylammonium bromide (CTAB) method. 50 mg DNA was used to construct the PacBio Sequel sequencing libraries, producing raw PacBio reads after sequencing. Paired-end libraries with insert sizes of 292, 319, 340, 394, 448, 514, 578, 812, and 904 bp were constructed using NEBNext Ultra II DNA Library Prep Kit for Illumina (NEB, USA). All the constructed libraries were sequenced on an Illumina HiSeq2500 platform (Illumina, USA) with a PE-100 module. The raw data were filtered by removing reads with more than 5% N or more than 40 bp low-quality bases (Q20). Moreover, only one copy of any duplicated paired-end reads was retained. The clean data was employed to correct the CANU-assembled genome.

All paired-end Illumina raw reads were subjected to the 21 k-mer frequency distribution analysis using Jellyfish (Version: 2.2.10) (Marcais and Kingsford, 2011). The result was fed to GenomeScope2 (Version: 2.0) (Ranallo-Benavidez et al., 2020) to estimate the genome size.

After automatic assembly was performed using CANU (Version: v1.8) (Koren et al., 2017) based on PacBio data with parameters “useGrid = true; minThreads=4; genomeSize=3000m; minOverlapLength = 700; minReadLength = 1000”, the assembled contigs were polished with clean NGS reads using Pilon (Version 1.23) (Walker et al., 2014). The polished contigs were further assembled using HERA (Du and Liang, 2019) with parameters “InterIncluded_Side=30000; InterIncluded_Identity=99; InterIncluded_Coverage=99; MinIdentity=97; MinCoverage=90; MinLength=5000; MinIdentity_Overlap=97; MinOverlap_Overlap=1000; MaxOverhang_Overlap=100; MinExtend_Overlap=500”.

The contigs were scaffolded with optical mapping using the SAPHYR optical mapping technology (BioNano Genomics). High molecular weight DNA with a fragment distribution greater than 150 kb was isolated using BioNano sample preparation kits (BioNano Genomics). Sequence-specific labeling of genomic DNA (gDNA) for BioNano mapping was prepared by Nicking, Labeling, Repairing, and Staining (NLRS). 600 ng of the isolated DNA was used for subsequent DNA nicking using Nt.BssSI (NEB) incubating for 2 hours at 37°C. The nicks were labeled according to the instructions provided with the BioNano Prep NLRS Labeling Kit (BioNano Genomics). The labeled DNA sample was loaded onto the Saphyr Chip nanochannel array (BioNano Genomics) and imaged using the Saphyr system and associated software (BioNano Genomics) according to the Saphyr System User Guide. The Sovle (Version: 3.1_08232017; BioNano Genomics) software package (parameters: -B 1 -N 2) was used to assemble the maps and construct scaffolds.

The software Redundans.py with parameters “–identity 0.55 –overlap 0.80 –noscaffolding –nogapclosing” was performed to resolve the redundancy in the genome first, and then the scaffolds were aligned all-to-all using BWA-MEM (Version: 0.7.12) (Li and Durbin, 2009). The coverage ratio was calculated as the length of synteny region/total length for an aligned length greater than 1 kb and identity above 96%. The sequences with more than 99% coverage ratio were filtered out.

After removing these heterozygous sequences, the remaining sequences were clustered using Hi-C data based on 3d-dna (Version: 180419) (Dudchenko et al., 2017). The Hi-C library was prepared by NowBio Biotechnology Co., Ltd (Yunnan, China), and then the library was sequenced on an Illumina NovaSeq platform. The clean Hi-C reads were mapped to the draft assembly with juicer (juicer_tools.1.7.6_jcuda.0.8.jar) (Durand et al., 2016). A candidate chromosome-scale assembly was generated automatically using a 3d-dna pipeline (Dudchenko et al., 2017). Manual review and refinement of the candidate assembly were performed in Juicebox Assembly Tools (Version 1.9.1) (Durand et al., 2016) for quality control and interactive correction. Then, the genome was re-assembled using 3d-dna (Dudchenko et al., 2017) according to manually adjust. With the modified 3d-dna and Juicebox workflow, 15 chromosomes were anchored. We performed BUSCO (v5.4.4) (Simao et al., 2015) assessments on the assembly using the embryophyte_odb10 lineage dataset (creation date: 2020-09-10, number of BUSCOs: 1,614).

### 2.2 RNA-seq

‘YK10’ leaves were collected at the shoot, mature, and late developmental stages. 3μg of total RNA per sample was used as input material for the RNA sample preparation. Beads with oligo (dT) were used to isolate poly(A) mRNA from total RNA. RNA sequencing libraries were constructed from these mRNA using the TruSeq RNA Sample Preparation Kit (Illumina, San Diego, USA). The resulting libraries were sequenced using the PE150 module of the Illumina platform. In addition, the Iso-Seq library for leaf tissue was prepared according to the Isoform Sequencing protocol (Iso-Seq) using the Clontech SMARTer PCR cDNA Synthesis Kit and the BluePippin Size Selection System protocol as described by Pacific Biosciences. Then, the library was sequenced on a PacBio Sequel System.

### 2.3 Repeats annotation

First, we searched for tandem repeats across the genome using Tandem Repeat Finder (TRF, Version: 4.09) (Benson, 1999). The transposable elements (TEs) in the genome were identified by combining homology-based and *de novo* approaches. For homolog-based prediction, known repeats were identified using RepeatMasker (Version: open-4.0.9) (Tarailo-Graovac and Chen, 2009) and RepeatProteinMask (Tarailo-Graovac and Chen, 2009) against Repbase (Jurka et al., 2005) (Repbase Release 20181026). For *de novo* prediction, LTR FINDER (Version: v1.07) (Xu and Wang, 2007) and LTRharvest in GenomeTools (Version: 1.5.10) (Ellinghaus et al., 2008; Gremme et al., 2013) were used to predict LTR retrotransposons, and the results were integrated using LTR_retriever (Version: v2.8) (Ou and Jiang, 2018). Furthermore, RepeatModeler (Version: 2.0; http://repeatmasker.org/) was also used to identify repeats from the assembled genome. The results of LTR_retriever and RepeatModeler were merged as a custom library and fed to RepeatMasker to predict TEs. The assembly index (LAI) and LTR insert time analyses were based on the LTR_retriever results (Ou and Jiang, 2018). The insertion time was calculated according to the formula Time=Ks/2μ (μ = 6.5×10^-9^ mutations per site per year) (Wang et al., 2020).

Finally, these TE sequences were classified according to their characteristics using TEsorter (https://github.com/zhangrengang/TEsorter) based on the REXdb database (viridiplantae_v3.0) (Neumann et al., 2019). For non-autonomous TEs that lack protein domains, such as SINEs, were classified according to their best-hit sequences using the method above. LTR-retrotransposon sequences encoding reverse transcriptase (RT) protein domains were extracted and used to construct a phylogenetic tree using FastTree (Price et al., 2010). The tree was created for each lineage of TEs separately. For rooting, we included ten sequences from other lineages. Ty3/gypsy Retand sequences were included as an outgroup for Ty1/Copia Angela and Ty1/Copia Ivana trees. Ty3/Gypsy Athila trees were rooted using Ty1/copia Angela.

### 2.4 Gene prediction, functional annotation, and evolution

We employed EVidence Modeler (EVM) (Haas et al., 2008) to consolidate RNA-seq-based, *de novo*, and homolog-based predictions into a final gene set. For RNA-seq-based gene prediction, the reads of Illumina transcriptome were cleaned with Trimmomatic (Bolger et al., 2014) and aligned to the genome with HISAT2 (Version: 2.2.0) (Kim et al., 2019). Alignments were assembled independently with StringTie (Version: 2.1.4) (Kovaka et al., 2019). For *de novo* gene prediction, the clean reads of Illumina transcriptome were assembled using Trinity (Version: v2.14.0) (Grabherr et al., 2011). Full-length transcriptomes were managed with the SMRT Analysis software suite (PacBio, Version: release_6.0.0.47841) and resulted in a set of full-length transcripts. RNA-seq assemblies and full-length transcripts were combined and further refined using PASA (Haas et al., 2008), and a high-quality training set was generated using the PASA assemblies. Then Augustus (Stanke et al., 2008) was performed with this training set. Three additional *ab initio* gene predictions, GlimmerHMM (Majoros et al., 2004), GENSCAN (Burge and Karlin, 1997), and SNAP (Korf, 2004) were also used. Protein sequences of *C. sinensis* var. *assamica* (CSA) (Xia et al., 2017) and *C. sinensis* var. *sinensis* (CSS) (Wei et al., 2018) were used for homolog-based gene annotation. After mapping the protein sequences to the ‘YK10’ genome using tblastn, protein-coding regions were obtained by extending 2,000 bp upstream and downstream of the matching DNA sequences. GeneWise (Birney and Durbin, 2000) was then used to predict gene structure within each protein-coding region. All lines of evidence were then fed to EVM using intuitive weight (RNAseq > cDNA/protein > *de novo* gene predictions). Finally, EVM models were updated with PASA. The genes were filtered if the proportion of repeats was more than 50% or the CDS was less than 300 nt.

Gene functions were assigned according to the best match alignment using eggNOG-mapper (Huerta-Cepas et al., 2017) against the eggNOG5.0 database. KEGG enrichment was performed using TBtools (Chen et al., 2020).

We downloaded and assembled the RNA sequencing data for the *C. japonica* cultivar ‘Jiangxue’ (PRJNA325385; (Li et al., 2016b). The data of three biological replicates for CK were used in our assembly. The transcriptome was *de novo* assembled using Trinity (Version: v2.14.0) (Grabherr et al., 2011) and filtered using CD-HIT-EST with parameter “-c 0.95” (Li and Godzik, 2006). Then, TransDecoder (Version: v5.5.0, https://github.com/TransDecoder/TransDecoder) was used to identify coding regions. Here, to maximize sensitivity for capturing ORFs, all ORFs were scanned for homology to known proteins dataset of ‘YK10’, TGY (CSS Chinese Oolong tea variety ‘Tieguanyin’) (Zhang et al., 2021), DASZ (an ancient tea tree) (Zhang et al., 2020b), and CON (wild oilseed *Camellia*, *C. oleifera* var. ‘Nanyongensis’) (Lin et al., 2022). The alternative splicing was filtered by CD-HIT-EST with the parameter “-c 0.9”. This step yielded 46,312 protein-coding genes and captured 87.7% complete orthologs (81.6% complete and single-copy orthologs and 6.1% complete and duplicated orthologs) in embryophyte_obd10 (Creation: 2020-09-10, number of BUSCOs: 1614) using BUSCO (Version: 5.4.2) (Simao et al., 2015).

The gene families cluster was performed with protein-coding genes of ‘YK10’, TGY, DASZ, CON, *C. japonica*, and *Actinidia chinensis* cultivar ‘Hongyang’ (Kiwifruit) (Yue et al., 2022) using OrthoFinder (Version: 2.5.4) (Emms and Kelly, 2019). The alignment of multiple single-copy orthologous genes and corresponding rooted species tree generating with OrthoFinder were fed to MCMCTREE in the PAML package (Version: 4.9j) (Yang, 2007) to infer the species divergence times. To estimate each species’ divergence time, the known divergence times between these species were collected from http://timetree.org. Based on the calculated phylogeny and divergence times, CAFE5 (Mendes et al., 2020) was applied to identify whether gene families had undergone expansion or contraction. The Gamma model with two gamma rate categories and the root equilibrium frequency with Poisson distribution were set.

All-to-all BLASTP analysis of protein sequences was performed within YK10, TGY, DASZ, and CON, respectively, using an e-value cutoff of 1e-10. Syntenic regions within each species were then identified using MCscanX (Wang et al., 2012) based on the all-to-all BLASTP results. Protein sequences of homologous gene pairs in the identified syntenic regions were first aligned using MUSCLE (Edgar, 2004), and the protein alignments were then converted to the CDS alignments. Finally, synonymous substitution rates (Ks) were calculated on these CDS alignments using KaKs_Calculator (Version: 1.2) (Zhang et al., 2006) with the NG method.

### 2.5 DNA sample preparation and sequencing for resequencing

486 *Camellia* accessions were obtained from the sampling place, containing 15 Other *Camellia* accessions (other species in *Camellia*), 21 ornamental *Camellia* accessions, 23 oilseed *Camellia* accessions, 54 Wild Tea accessions, 20 Hybrid accessions (*C. sinensis* var. *assamica* × *C. sinensis* var. *sinensis*), 246 CSA accessions (*C. sinensis* var. *assamica*) and 107 CSS accessions (*C. sinensis* var. *sinensis*). In addition, 139 accessions (1 oilseed *Camellia* accession, 13 Wild Tea accessions, 5 Hybrid accessions, 37 CSA accessions, and 83 CSS accessions) in a previous paper (Wang et al., 2020) were also used for resequencing analysis. Young leaves were collected from the plants and snap-frozen in liquid nitrogen. Total DNA was extracted with the DNAsecure plant kit (Tiangen, Beijing). 2 µg genomic DNA from each accession was used to construct a sequencing library following the manufacturer’s instructions using NEBNext Ultra DNA Library Prep Kit (NEB, USA). Paired-end sequencing libraries with an insert size of approximately 400 bp were sequenced on an Illumina NovaSeq 6000 sequencer at Novogene (Beijing, China). Paired-end resequencing reads were filtered using NGSQCToolkit_v2.3.3 (Patel and Jain, 2012), removing reads containing adapter or poly-N and low-quality reads (reads with >40% bases having Phred quality ≤ 20) from the raw data. Reads shorter than 70bp were discarded, and the yielded clean data were used for downstream analyses.

### 2.6 Variation calling and annotation

Paired-end reads were mapped to the ‘YK10’ genome using bwa-mem2 (Version: 2.0pre1) (Md et al., 2019) with the default parameters. SAMtools (Version: 1.3.1) (Li et al., 2009) converted mapping results into the BAM format. After sorting the mapping results, duplicated reads were marked with the Picard package (picard.sourceforge.net, Version: 2.1.1). The reads around indels were then realigned using Genome Analysis Toolkit (GATK, version 3.3-0-g37228af) (McKenna et al., 2010).

The variation detection followed the best practice workflow recommended by GATK (McKenna et al., 2010). In brief, the variants were called for each accession with GATK HaplotypeCaller (McKenna et al., 2010) with a joint genotyping step. In the filtering step, the SNP filter expression was set as “QD < 2.0 || MQ < 40.0 || FS > 60.0 || SOR > 5.0 || MQRankSum < -12.5 || ReadPosRankSum < -8.0 || QUAL < 30”, and the Indel filter expression was set as “QD < 2.0 || ReadPosRankSum < -20.0 || InbreedingCoeff < -0.8 || FS > 200.0 || SOR >10.0 || QUAL < 30”. Only insertions and deletions shorter than or equal to 40 bp were considered. Indels and SNPs with non-biallelic were removed, which yielded the basic set. SNPs with MAF < 0.05 and >50% missing calls were further removed for phylogenetic tree structure, population structure analyses, and IBS calculation (the core set).

SNPs and Indels annotation were performed according to the ‘YK10’ genome using ANNOVAR (Version: 2015-12-14) (Wang et al., 2010). The coverage of each accession against each chromosome of the ‘YK10’ genome was counted based on the aligned BAM file using SAMtools (Version: 1.3.1) (Li et al., 2009). A 100 kb sliding window approach was applied to quantify genetic differentiation (*F_ST_*) and polymorphism levels (π, pairwise nucleotide variation as a measure of variability) using VCFtools (v0.1.16) (Danecek et al., 2011).

### 2.7 Population genetics analysis

Whole-genome SNPs were used to construct the ML (Maximum likelihood method) phylogenetic tree with 100 bootstraps using SNPhylo (Version: 20140701) (Lee et al., 2014). *C. cuspidata* (KM6) was used to provide outgroup information. The iTOL tool (http://itol.embl.de) was used to color the phylogenetic tree.

SNPs in linkage disequilibrium (LD) were filtered using PLINK (Version v1.90b3.38) (Purcell et al., 2007) with a window size of 50 SNPs (advancing 5 SNPs at a time) and an *r* ^2^ threshold of 0.5. Population structure was analyzed using ADMIXTURE (Version: 1.3) (Alexander et al., 2009) with a block-relaxation algorithm. To explore the convergence of individuals, we predefined the number of genetic clusters K from 2-8 and ran the cross-validation error (CV) procedure with default methods and settings.

### 2.8 Clustering and discretization

Genetic distances among and within each accession were calculated based on LD pruned SNPs using PLINK with the formulation 1-IBS, where IBS is identity by state. The CMDSCALE function in R was used to calculate eigenvectors (Mardia, 1978) based on the distance matrix. Formal clustering with the PAM method and filtering based on each cluster’s silhouette scores computed by the DISCRETIZE algorithm were performed with the IBS distance matrix using an available R script (https://github.com/grafau/discretize), which was previously described (Gutaker et al., 2020). Clustering and discretization were carried out independently for clusters k, ranging from 2 to 9. The remaining 443 accessions in the corresponding group with k=5 were selected and filtered to remove outliers based on the phylogenetic tree. The filtered accessions were used for subsequent analysis.

### 2.9 Relationship inference

Relationships between each accession were verified using KING (Version: 2.2.5) (Manichaikul et al., 2010) based on the basic set of SNPs. Close relatives were inferred based on the estimated kinship coefficients as shown in the following algorithm: an estimated kinship coefficient range >0.354, [0.177, 0.354], [0.0884, 0.177] and [0.0442, 0.0884] correspond to duplicate, 1st-degree, 2nd-degree, and 3rd-degree relationships, respectively. The duplicate accessions were removed from subsequent group analysis.

### 2.10 Genome scanning for selective sweep signals

RAiSD (Raised Accuracy in Sweep Detection, Version 2.9) (Alachiotis and Pavlidis, 2018) was used to detect signatures of selective sweeps based on the μ statistics in ornamental *Camellia*, oilseed *Camellia*, Wild Tea, CSA 1, CSA 2, and CSS group, respectively. The significant threshold for μ statistic score was set as top 0.5%.

### 2.11 Linkage disequilibrium

LD was calculated using PopLDdecay (Version: v3.41) (Zhang et al., 2019). The pairwise *r* ^2^ values within and between different chromosomes were calculated. The LD for each group was calculated using SNP pairs only from the corresponding group.

### 2.12 Demographic history reconstruction using the PSMC approach

The Pairwise Sequentially Markovian Coalescent (PSMC) model (Sun and An, 2005) was used to reconstruct the demographic history of each tea accession. Parameters were set as follows: -N25, -t15, -r5 and -p ‘4 + 25*2 + 4+6’. The estimated time to the most recent common ancestor (TMRCA) was given in units of 2N_0_ time, and the relative population size (N_e_) at state *t* was scaled to N_0_ (the present effective population size). Then, the neutral mutation rate (6.1 × 10^-9^ mutations per site per year) was used to scale the TMRCA and N_e_ values into chronological time. For plotting, accessions were grouped by their populations.

### 2.13 Demographic history inference using SMC++

SMC++ (version: v1.15.4.dev16+g72ea2e2) (Terhorst et al., 2017) was employed to infer population size histories and split times between the CSA 2 and CSS groups. The analysis was performed by testing the treatment of runs of homozygosity longer than 50, 100, and 200 kb as missing or not (no mask). A mutation rate of 6.1 × 10^-9^ mutations per site per year was used to convert the scaled times and population sizes into real times and sizes.

### 2.14 Analysis of self-organizing maps machine learning

After clustering, discretization, and removing duplicate accessions, the CSA 1 (54), CSA 2 (98), and CSS (176) tea accessions were analyzed using self-organizing maps (SOM) machine learning. SNPs with MAF > 0.05 and no missing genotypes were selected. SNPs in linkage disequilibrium were filtered using PLINK (Version v1.90b3.38) (Purcell et al., 2007) with parameter “–indep-pairwise 50 1 0.5”. Then, SNP-genotypes were coded with the integers: 0 - homozygous major alleles genotype, 1 - heterozygous alleles genotype, and 2 - homozygous minor allele genotype. The datasets were feature-centralized and clustered using SOM machine learning (Wirth et al., 2011). SOM was implemented in “oposSOM” R package (Löffler-Wirth et al., 2015).

### 2.15 Treemix

We estimated admixture graphs in SOM-defined tea clusters using TreeMix (Version: 1.12) (Pickrell and Pritchard, 2012), employing a Maximum Likelihood (ML) method based on a Gaussian model of allele frequency change. Migration events (*m*) were set from 1 to 5. The bootstrap values were calculated with 1,000 replicates, using the Wild Tea group as the root. Each migration event was run ten times with a random seed. The optimal number of migration edges was estimated using an R package “OptM” (Version: v0.1.6) (Fitak, 2021). Node robustness was estimated with 100 bootstrap replicates and plotted using the treemix.bootstrap function in BITE (Version: v1.2.0008) (Milanesi et al., 2017).

### 2.16 Environmental niche modeling

We compiled 5,415 occurrence records for *C. sinensis* (L.) Kuntze from the Global Biodiversity Information Facility (GBIF, www.gbif.org). Duplicate records and those lacking location data were omitted, and longitude and latitude were supplemented according to the locality. The filtered occurrences were further thinned to ensure records were at least 50 km apart using the R package “spThin” (Aiello-Lammens et al., 2015), resulting in 459 records. Rasters of 19 bioclimatic variables at 2.5-minute resolution for Last Glacial Maximum (LGM, ca. 21 ka, v1.2b), early-Holocene (EH, Greenlandian, 11.7-8.326 ka, v1.0), mid-Holocene (MH, Northgrippian, 8.326-4.2 ka, v1.0), late-Holocene (LH, Meghalayan, 4.2-0.3 ka, v1.0) and Current (1979-2013, Anthropocene, v1.2b) paleoclimate data were obtained from PaleoClim (Brown et al., 2018). The ecological niche models were run over all combinations of the defined settings and evaluated with cross-validation using the R package “ENMeval” (Muscarella et al., 2014). The setting of LQHP_2 (here: L: Linear features; Q: Quadratic features; H: Hinge features; P: Product features; 2: Regularization multiplier value), LQHPT_3 (T: Threshold features), LQHPT_2.5, H_2.5 and L_3 were used to measure variable importance for each period. Habitat suitability projections for the four periods were generated using MaxEnt (Version: 3.4.4) (Phillips et al., 2017) with ENMeval results, ten subsample replicated runs, and a 30% random test.

### 2.17 Pan-genome construction

The accessions used for pan-genome analysis were selected according to manual screening and SVCollector (Ranallo-Benavidez et al., 2021). The manual screening followed these principles: (1) remove accessions with sequencing depth lower than 7 ×; (2) remove hybrid accessions; (3) remove the potentially wrong accessions whose position in the SNPs’ phylogenetic tree was incongruent with the accession information; (4) just keep one accession if there were multiple cultivars; (5) retain one accession per species in a clade of the SNPs’ phylogenetic tree for ornamental *Camellia*, oilseed *Camellia*, and Wild Tea groups. After combining accessions selected by the two methods, accessions were further removed if they were not in the accession pool, resulting in clustering, discretization, and removing duplicate accessions. After the above filtering step, sequences of 206 tea accessions were used for the pan-genome construction.

Raw reads of selected accessions were processed to remove duplicated reads using Nubeam-dedup (Dai and Guan, 2020). Then, the adapters and low-quality sequences were trimmed using Trimmomatic (Version: 0.39) (Bolger et al., 2014) with parameters ‘SLIDINGWINDOW:4:20 MINLEN:50’. The final cleaned reads of each accession were *de novo* assembled using Megahit (Version: v1.2.9) (Li et al., 2016a) with default parameters. The non-reference sequences were identified according to the previous description (Gao et al., 2019). In brief, the assembled contigs with lengths longer than 500 bp were selected and aligned to ‘YK10’ genomes using Mummer (Version: 4.0.0beta2) (Delcher et al., 2003). If the continuous alignment was longer than 300 bp with sequence identity higher than 86%, and the continuous unaligned regions were longer than 500 bp, then the unaligned regions were extracted as unaligned sequences. These unaligned sequences and other unaligned contigs were then searched against the GenBank nucleotide database using blastn (Version: 2.9.0+) (Camacho et al., 2009). Sequences with the best hits outside the green plants or covered by known plant mitochondrial or chloroplast genomes were removed. The cleaned non-reference sequences from all accessions were combined and then processed to remove redundant sequences using CD-HIT (Version: 4.8.1) (Li and Godzik, 2006) with an identity threshold of 90%. The resulting non-redundant sequences and the reference ‘YK10’ genome were merged as the pan-genome.

### 2.18 Annotation of the pan-genome

A custom repeat library was constructed by screening the pan-genome using EDTA (Version: v1.9.6) (Ou et al., 2019) and used to screen the non-reference genome to identify repeat sequences with RepeatMasker (Version: 4.1.2-p1) (Tarailo-Graovac and Chen, 2009). Protein-coding genes were predicted from the repeat-masked non-reference genome using BRAKER (Version: 2.1.6) (Brůna et al., 2021) with two lines. One of the lines was running BRAKER with RNA-Seq data. RNA-seq data included Illumina transcriptome for leaves sequencing in our paper and reads of 40 tea samples downloaded from NCBI (**Supplementary Table S1**). The reads were cleaned using Trimmomatic (Version: 0.39) (Bolger et al., 2014) with parameters ‘SLIDINGWINDOW:4:15 MINLEN:70’ and aligned to the pan-genome with HISAT2 (Version: 2.2.0) (Kim et al., 2019). Then, the alignments were converted to a hint file for AUGUSTUS in gff format. All gff files were merged and fed to BRAKER. Another line was running BRAKER with OrthoDB Viridiplantae protein database. The results were then combined using TSEBRA (https://github.com/Gaius-Augustus/TSEBRA). The genes were filtered if the proportion of repeat sequences in gene sequences was greater than 50% or the CDS length was less than 300 nt.

Gene functions were assigned according to the best match using blastp against KEGG databases. InterPro functional analysis and Gene Ontology IDs were obtained using InterProScan (Zdobnov and Apweiler, 2001). The pathway to which the gene might belong was derived from the matching genes in KEGG.

### 2.19 PAV analysis

Sequencing reads from each accession were aligned to the pan-genome using Bowtie2 (Version: 2.4.4) (Langmead and Salzberg, 2012) with “–end-to-end –sensitive” parameters. The gene presence/absence variation was characterized with the SGSGeneLoss package (Version: v0.1) (Golicz et al., 2015). For a specific gene within a given accession, if less than 20% of its exon regions were covered by at least two reads (minCov = 2, lostCutoff = 0.2), this gene was treated as absent in that accession; otherwise, it was determined as present. A maximum-likelihood phylogenetic tree was constructed based on the PAVs with 1,000 bootstraps using IQ-TREE (Version: 1.6.12) (Nguyen et al., 2015) with *C. costei* (KM7) as outgroup. Population structure was determined using ADMIXTURE (Version: 1.3) (Alexander et al., 2009). Principal component analysis was performed with TASSEL5 (Bradbury et al., 2007).

The presence frequencies within each pair of groups (ornamental *Camellia* versus oilseed *Camellia* and CSA versus CSS) were derived to identify genes under selection. The significance of the difference in the presence frequencies for each gene between the two compared groups was determined using Fisher’s exact test. The raw P values of all genes in each comparison were corrected using a false discovery rate (FDR). Genes under selection were identified with significantly different frequencies (FDR < 0.001 and fold change >2). KEGG enrichment analysis was performed using TBtools (Chen et al., 2020).

### 2.20 Pan-genome modeling

Pan-genome and core-genome size curves were fitted using the nlsLM function in the R package minpack.lm according to the steps (Hurgobin et al., 2018). The combinations of genomes were obtained according to the formula: 206!/(n!(206-n))!, n = [1,206]. The pan-genome size was modeled using the power law regression: y = Ax^B^ + C, and the core genome size was modeled using the exponential regression y = Ae^Bx^ + C (Tettelin et al., 2005; Zhao et al., 2014).

## 3 Results

### 3.1 ‘Yunkang 10’ genome assembly and *Camellia* genomic variant

**Figure 1:**
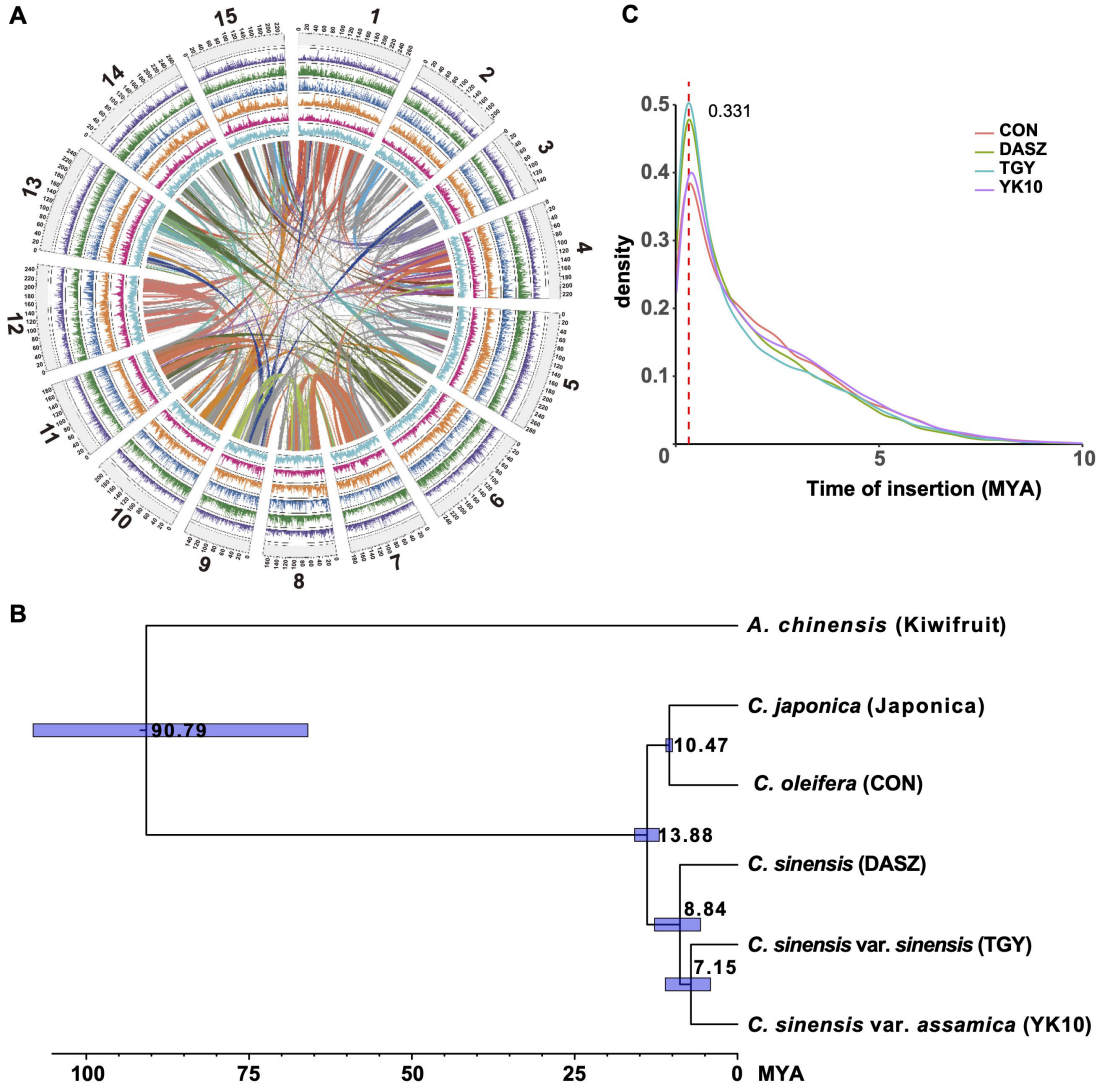
Genome assembly and evolution of the ‘YK10’. **(A)** The genome information of ‘YK10’. Representatives from outside to inside: chromosomes (light gray), LTR number (purple), gene number (green), TRF number (blue), Copia number (orange), Gypsy number (red), and GC content (cyan). **(B)** Phylogenetic tree and divergence time of ‘YK10’ and five plant species. The phylogenetic tree was generated from single-copy orthologs using the maximum-likelihood method. Blue blocks show the 95% highest posterior density (HPD) of divergence time. **(C)** Distribution of LTR-RT insertion time for different tea trees based on LTR_retriever’s result. ‘YK10’: CSA cultivar ‘Yunkang10’; TGY: CSS Chinese Oolong tea variety ‘Tieguanyin’; CON: wild oilseed *Camellia* (*C. oleifera* var. ‘Nanyongensis’); DASZ: an ancient tea tree; Kiwifruit: *Actinidia chinensis* var. ‘Hongyang’.

‘Yunkang10’ (‘YK10’) is a CSA tea tree cultivar (2n=2x=30). Its estimated genome size was ~3.05 Gb using *k*-mer (*k* = 21) analysis with 670.98 Gb Illumina sequencing data (**Supplementary Figure S1A**, **Supplementary Table S2**). Due to the high genome heterozygosity level of 2.99%, we obtained an initial 5.54 Gb ‘YK10’ genome assembly from 318.86 Gb PacBio single-molecule sequencing data (**Supplementary Table S3**; contig N50 = 148,300 bp). Then, the contigs were scaffolded using 574.98 Gb optical mapping data (**Supplementary Table S4**). This step increased the contig N50 size to 822,186 bp. After removing redundancy and heterozygous sequences in the genome, we attained an assembly of 3.35 Gb with a contig N50 of 816,948 bp. Further, we clustered it into 15 chromosome-scale scaffolds using Hi-C data (**Figure 1A**, **Supplementary Figure S1B**, **Table 1**, **Supplementary Tables S5** and **S6**). Our assembly captured 94.7% (1,529 of 1,614) of the complete core embryophyta genes (**Supplementary Table S7**), and the LTR assembly index (LAI) score of 11.94 suggested high completeness (**Supplementary Table S8**).

**Table 1:** Summary of genome assembly and annotation.

Assembly	
Assembled genome size (bp)	3,346,234,254
Genome-sequencing depth (×)	
NGS	200.29
PacBio	95.18
BioNano	171.63
Hi-C	97.00
Number of contigs	6,205
N50 of contigs (bp)	816,948
No. of chromosome	15
GC content of the genome (%)	37.13
Completeness evaluation	
BUSCO (Based on embryophyta_odb10 database)	94.7%
Annotation	
Percentage of repeat sequences (%)	84.62
Repeat sequence length (bp)	2,831,450,703
No. of predicted protein-coding genes	42,536
No. of predicted transcripts	54,598
Average gene length (bp)	6,174.33
Average mRNA length (bp)	1,675.93
Average CDS length (bp)	1,251.11
Average protein length (aa)	417.03
BUSCO of genes (Based on embryophyta_odb10 database)	79.5%

We predicted 42,536 protein-coding genes (54,598 transcripts) following homology and *de novo* methods (**Supplementary Tables S9** and **S10**; 79.5% of core BUSCO genes). In total, 35,328 (83.05%) predicted genes had functional annotation in public databases (**Supplementary Table S11**). The species phylogeny using single-copy orthologous genes of reported *Camellia* genomes (**Figure 1B**, **Supplementary Table S12**) revealed an estimated divergence time of ~7.15 million years ago (MYA) between ‘YK10’ and ‘Tieguanyin’. The divergence time between *C. japonica* and *C. oleifera* var. ‘Nanyongensis’ was estimated at ~10.47 MYA. Tea trees diverged from other *Camellia* species at ~13.88 MYA. Whole genome duplication (WGD) analysis indicated that YK10 (and other *Camellia* plants) had experienced the same genome duplication event, in which the Ks peak was 0.386-0.414 (**Supplementary Figure S2**). 3,919 and 2,158 gene families of ‘YK10’ underwent expansion and contraction, respectively (**Supplementary Figure S3**), with 757 exhibiting significant expansion and 178 showing significant contraction. The significantly expanded genes were related to phenylpropanoid biosynthesis, glycan and glycosaminoglycan metabolism, ubiquinone and other terpenoid-quinone biosynthesis, and so on (**Supplementary Table S13**). Five gene families, including 26 *NUCLEOTIDE-BINDING SITE AND LEUCINE-RICH REPEAT* (*NBS-LRR*) genes, also underwent significant expansion. In comparison, the significantly contracted genes were related to monoterpenoid biosynthesis, metabolism of terpenoids and polyketides and others (**Supplementary Table S13**).

We predicted ~2.83 Gb repetitive elements in the ‘YK10’ genome (**Table 1**, **Supplementary Table S14**), among which 82.64% repeats were transposable elements (TEs; **Supplementary Table S15**). Long-terminal repeat (LTR) retrotransposons represented 58.48% of the genome, with Ty3/gypsy Ogre elements being the major lineage (10.08%, **Supplementary Table S15**). We showed a universal distribution of major TE families across the genome (**Supplementary Figure S4**) and analyzed the divergence of the reverse transcriptase sequences of different TE lineages (**Supplementary Figure S5**). Notably, Athila elements were evolutionarily young, but Angela and Ivana were comparably more ancient (**Supplementary Figure S5**). The LTR retrotransposon burst event in ‘YK10’ was consistent with other reported genomes (**Figure 1C**), around 0.331 MYA.

**Figure 2:**
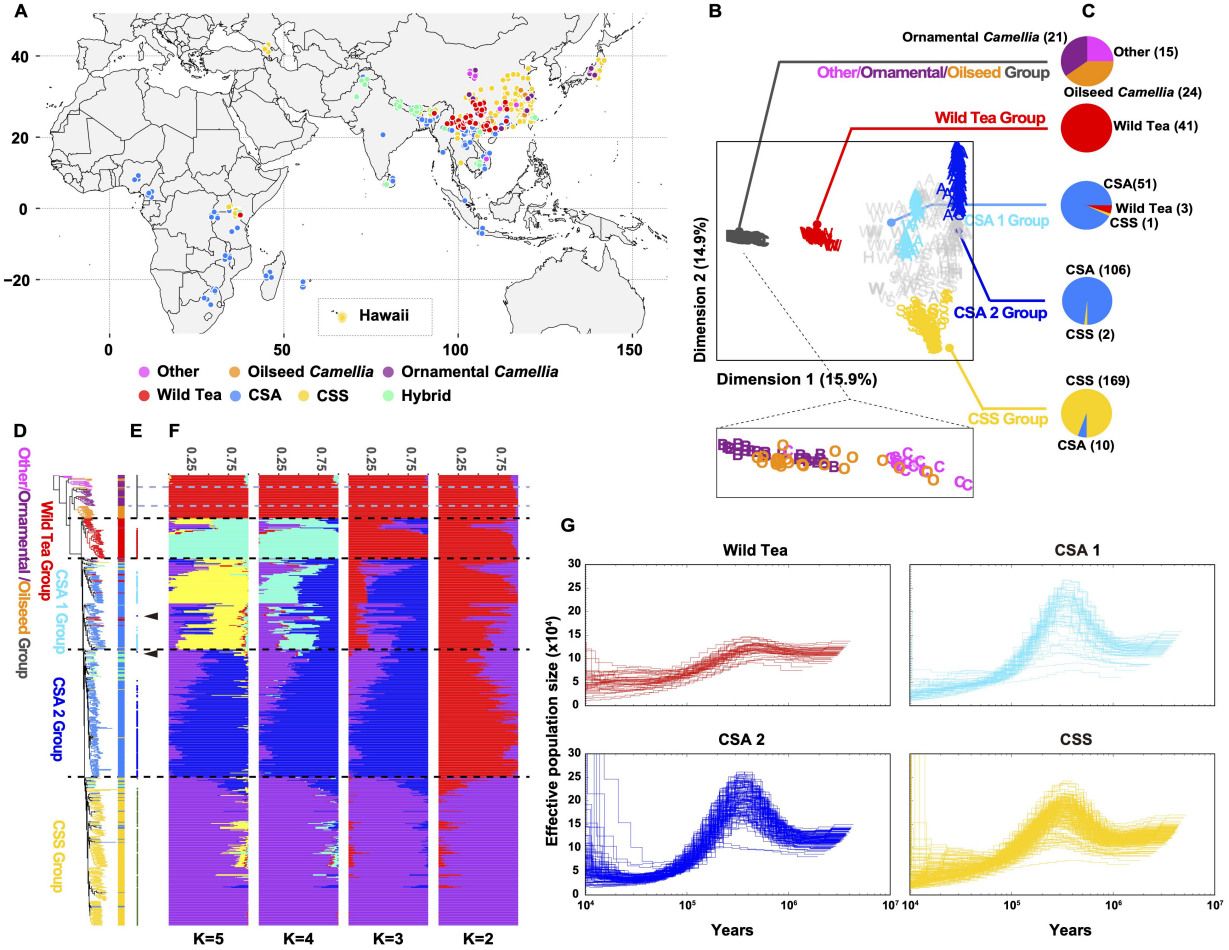
Subpopulations of tea accessions. **(A)** Geographic distribution of the re-sequenced *Camellia* accessions. **(B)** All tea accessions projected onto the first two dimensions after multidimensional scaling of genomic distances. The tea accessions’ genotypes were clustered using k-medoids (k = 5 subpopulations) and filtered using silhouette parameters, which resulted in k_d_=5 discrete subpopulations (colored labels, Other/ornamental/oilseed *Camellia*, Wild Tea, CSA 1, CSA 2 and CSS groups). **(C)** Pie charts represent the tea accession compositions of each discrete subpopulation. **(D)** Maximum likelihood phylogenetic tree of tea accessions inferred from the whole-genome SNPs with 100 nonparametric bootstraps (see **Supplementary Figure S7** for bootstrap values as blue circles). *C. cuspidata* (KM6) was used as an outgroup. **(E)** Signs represent the position on the phylogenetic tree for selected accessions of **(B)**. Arrows indicate outlier the accession (re166 and re176). **(F)** Population structure of major tea categories estimated by ADMIXTURE. Each color represents one ancestral population. Each accession is represented by a bar, and the length of each colored segment in the bar represents the proportion contributed by that ancestral population. **(G)** PSMC-inferred population history of Wild Tea, CSA 1, CSA 2, and CSS group. Each line represents an accession.

We collected 486 *Camellia* accessions worldwide and generated whole-genome sequencing data at an average depth of 18.28×, and we also included 139 accessions from a previous study (Wang et al., 2020) to maximize the genetic diversity for population genomic analyses (**Figure 2A**, **Supplementary Table S16**). The mean mapping rate of these reads to the ‘YK10’ reference genome was ~98.63% (**Supplementary Table S17**), and the properly paired mapping rate was 49.99-93.89% and showed significant difference among groups (**Supplementary Figure S6**, **Supplementary Table S17**). After applying filtering criteria to the called variants, we identified 651,670,193 single-nucleotide polymorphisms (SNPs), among which 80,356,229 SNPs had minor allele frequencies (MAF) more than 0.05 and <50% missing calls (**Supplementary Table S18**). About 91.87% of SNPs were intergenic. The nonsynonymous-to-synonymous substitution ratio for the SNPs in the coding regions was 1.419. We also found 3,727,828 indels (< 40 bp), 84.30% of which are in the intergenic regions and 0.83% in the coding regions. An estimated 72.82% of indels in the coding regions could cause frameshift mutations (**Supplementary Table S18**).

### 3.2 *Camellia* population structure

We constructed a maximum likelihood (ML) phylogenetic tree using *C. cuspidata* as an outgroup to investigate the phylogenetic relationships among *Camellia* accessions. We found that many *Camellia* species in the section *Theopsis* of subg. *Thea* (hereafter termed ‘Other’) were closer to the phylogenetic tree root (**Supplementary Figure S7**). In addition, the ornamental *Camellia* species form two polyphyletic clusters. The oilseed *Camellia* species also did not cluster together. This result suggested rampant genetic introgression with congeners or potential misclassification of accessions based on morphology during sample collection. In the section *Thea* of subgenus *Thea*, wild tea tree species, CSA, and CSS accessions formed three independent clades, with CSA and CSS being sister clades.

To identify discrete subpopulations (k_d_) of *Camellia* accessions, we clustered all accessions based on genomic distances by partitioning around medoids (PAM) and a silhouette-based procedure (Gutaker et al., 2020). This discretization procedure removed genetic gradients between subpopulations (**Figures 2B, C**), corresponding to the removal of accessions with mixed genetic ancestry assessed by ADMIXTRUE (**Figures 2D-F**, **Supplementary Figure S8**). In the end, we obtained five discrete subpopulations: The base group containing Other/ornamental/oilseed *Camellia* species, a second group containing wild tea trees (hereafter Wild Tea), the third and fourth groups containing CSA accessions (hereafter CSA 1 and 2), and the last group containing CSS accessions (hereafter CSS).

Furthermore, we removed the clonal accessions according to a KING-robust kinship criterion (> 0.354; **Supplementary Figure S9**). In our sample collections, first-degree relationships existed in many *Camellia* plants (KING-robust kinship value: 0.177 - 0.354). For example, the ornamental *Camellia* cultivars ‘Huamudan’ (KM9) and ‘Qiumudan’ (KM10) with distinct floral presentations may result from a single hybridization event. The Chinese CSS cultivar pairs ‘Fujian Shuixian’ (Per13-3) and ‘Shuixian’ (re024), the Malawian CSA cultivar pairs ‘SFS 371’ (re313) and ‘PC81’ (re314) with first-degree relationships showcased the hybridization history of tea breeding worldwide. We deduced that the Japanese tea accessions re094, re095, and re098 were offspring of the cultivar ‘Yabukita’, representing about 80% of the total cultivated areas in Japan. Previous research showed that ‘Fuyun 6’ (re035) was the offspring of ‘Fuding Dabai’ and one unknown CSA accession (Zhang et al., 2021). Our result revealed the identity of the unknown parent as ‘Zhongye 2’ (Per15-3), which originated from Fengqing County of Yunnan Province, China.

Characterization of the linkage disequilibrium (LD, expressed as *r* ^2^) pattern showed that the LD decay for all tea tree subpopulations was very rapid. The LD reached half the maximum average *r* ^2^ at a distance of 400 bp for the CSA 1 group, 100 bp for the CSA 2 group, and 100 bp for the CSS group. In comparison, the LD reached half of the maximum *r* ^2^ at a distance of 11.7 kb for ornamental *Camellia*, 3.4 kb for oilseed *Camellia*, and 400 bp for the Wild Tea group (**Supplementary Figure S10**).

### 3.3 Demographic history of CSA and CSS

We applied the pairwise sequentially Markovian coalescent model (Li and Durbin, 2011) to analyze the *Camellia* groups (**Figure 2G**). The results were scaled to real time, assuming a neutral mutation rate of 6.1 × 10^-9^ nucleotides per year. Almost all groups experienced a steady decline in effective population size (*N_e_*) from the highest point at 300-500 thousand years ago (Kya) to the nadir at 10-30 Kya. The Wild Tea group had a smaller *N_e_* at the highest point. We also explored the population split between CSA and CSS populations with unphased SNP data using SMC++ (Terhorst et al., 2017). This approach revealed that these two groups diverged at about 11 Kya (**Supplementary Figure S11**).

### 3.4 Trait selection signatures for ornamental, oilseed, and tea trees *Camellia*

**Figure 3:**
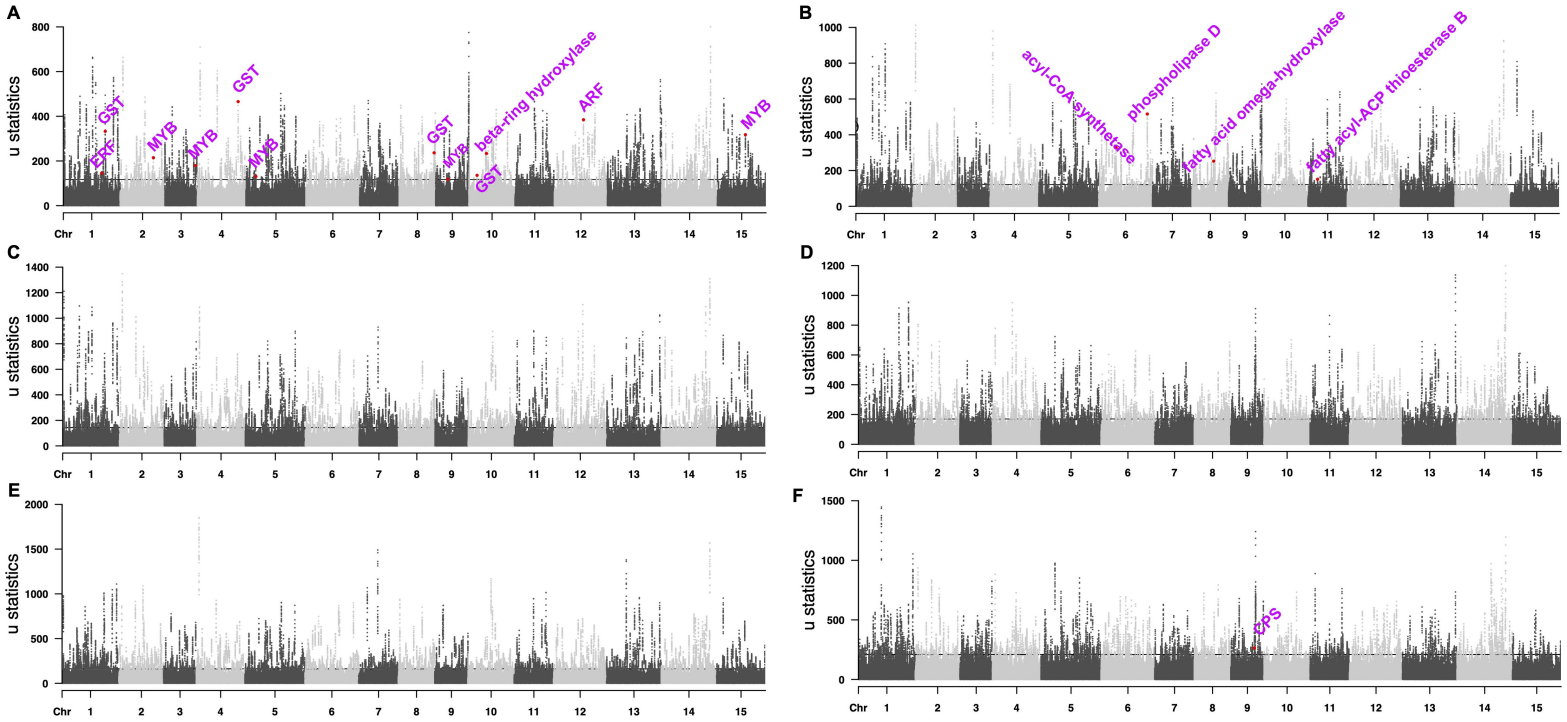
Manhattan plots of selected sweep signals. **(A)** ornamental *Camellia*. **(B)** oilseed *Camellia*. **(C)** Wild Tea. **(D)** CSA 1. **(E)** CSA 2. **(F)** CSS. The dashed lines mark the regions at the top 0.5%.

We used the top 0.5% RAiSD μ statistic score (Alachiotis and Pavlidis, 2018) to screen potential selective sweep signals in ornamental *Camellia* and identified 1,457 protein-coding genes in the selective sweep regions (**Figure 3A**, **Supplementary Data S1**). Gene function analyses revealed that these genes were enriched in plant secondary metabolism pathways (**Supplementary Table S19**). Lots of candidate genes were involved in flower development (**Figure 3A**, **Supplementary Data S1**), such as genes encoding auxin response factor protein (ARF, *CaS12G016450*), MYB transcription factor protein (*CaS02G017990, CaS03G017040, CaS05G008850, CaS09G008780*, and *CaS15G015140*), glutathione S-transferase (GST, *CaS01G024940, CaS04G022330, CaS08G023030*, and *CaS10G007120*), beta-ring hydroxylase protein (*CaS10G014230*) and ethylene-responsive transcription factor protein (*CaS01G023240*).

We also identified 1,714 protein-coding genes in the selective sweep regions for oilseed *Camellia*, among which many genes may help the increased *de novo* fatty acid (FA) biosynthesis (**Figure 3B**, **Supplementary Data S1**), e.g., phospholipase D (PLD, *CaS06G030600*), acyl-CoA synthetase (*CaS06G009730*), fatty acid omega-hydroxylase proteins (*CaS08G012810*, *CaS08G012820*), and fatty acyl-ACP thioesterase B protein (*CaS11G004150*).

In addition, we identified more genes in the selective sweep regions in the Wild Tea group (2,289) and the CSA groups (2,147 and 2,477) than in the CSS group (1,858; **Figures 3C-F**, **Supplementary Data S1**). The stilbenoid, diarylheptanoid, and gingerol biosynthesis pathway was enriched in all tea groups, which agreed with the selection of tea for a beverage. The selected genes in CSA groups contained members related to environmental adaptation and plant-pathogen interaction (**Supplementary Table S19**). Selective sweep genes in the CSA2 group also participated in the metabolism of terpenoids and polyketides, as well as monoterpenoid and brassinosteroid biosynthesis. By contrast, the CSS group had selective sweep genes related to flavonoid biosynthesis, diterpenoid biosynthesis, alpha-linolenic acid metabolism, and beta-alanine metabolism (**Supplementary Table S19**). In addition, we found a gene (*CaS09G017690*) encoding copalyl-diphosphate synthase (CPS) protein in the selective sweep region of the CSS group (**Figure 3F**), which may be related to dwarf phenotype (Guo et al., 2020).

### 3.5 Subpopulation structure in tea tree *Camellia*

**Figure 4:**
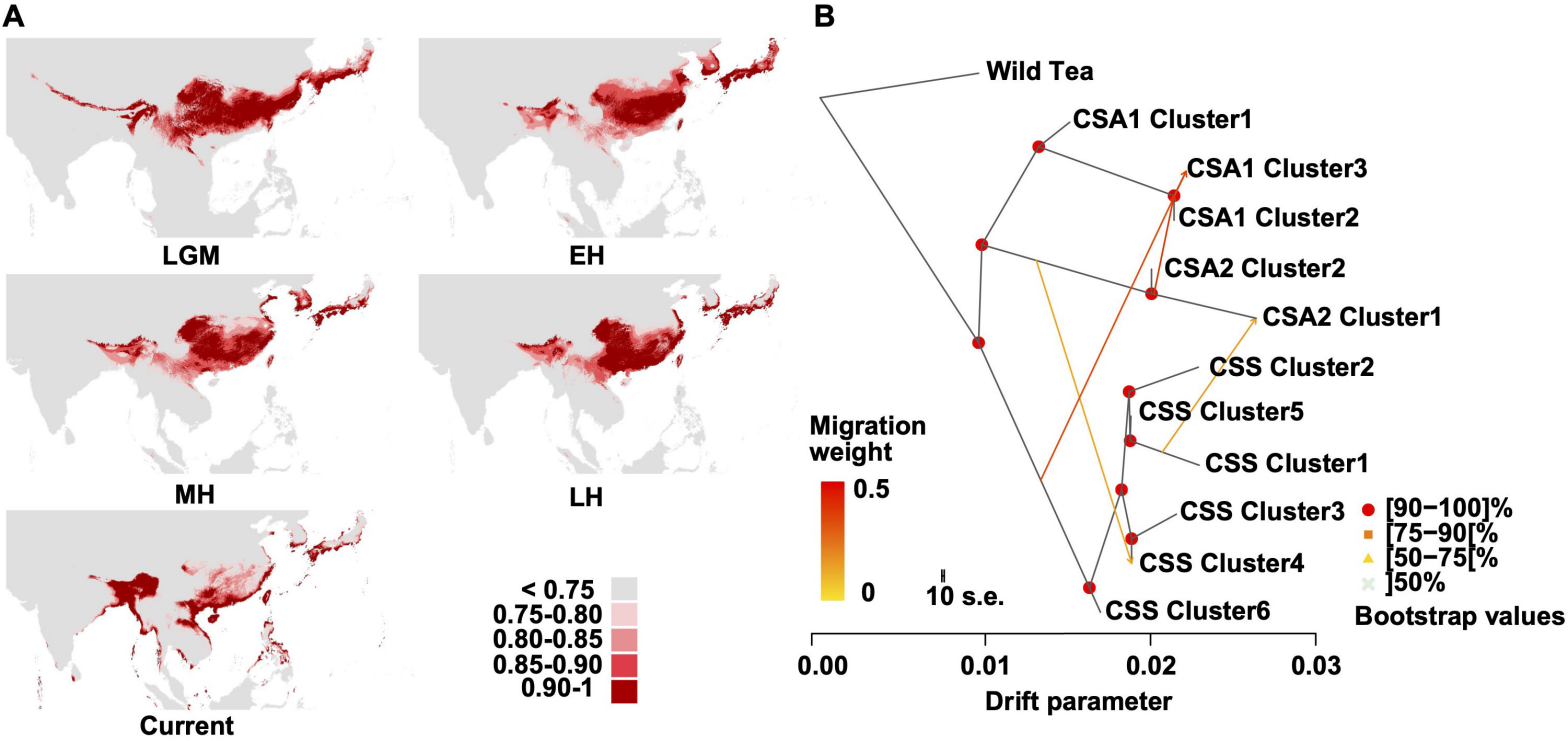
Environmental niche modeling and TreeMix analysis. **(A)** Predicated distributions of *C. sinensis* (L.) Kuntze based on ecological niche modeling during LGM (Last Glacial Maximum, ca. 21 ka), EH (EH (early-Holocene, 11.7-8.326 ka)), MH (mid-Holocene, 8.326-4.2 ka), LH (late-Holocene, 4.2-0.3 ka) and Current (1979 – 2013) period. Areas in different colors indicate the various probabilities (0-1) of suitable habitats. **(B)** The gene flow among the CSA and CSS clusters (m=4). The direction of each arrow represents the direction of gene flow. The Wild Tea group was used for root.

Ecological niche modeling revealed a significant suitable habitat (suitability score >0.75) for tea trees in the Southern regions of East Asia and Southeast Asia from the Last Glacial Maximum (LGM) to the Holocene period, consistent with the collected records from GBIF (**Figure 4A**, **Supplementary Figure S12**, **Supplementary Table S20**). It is also apparent that the suitable habitat gradually separated into two smaller regions since the Late Holocene. The west covers the Eastern Himalayan region and the Ganges Basin, whereas the east mainly covers South China. This result corresponds to the sister relationship between CSA and CSS (**Figure 2D**), hinting at the parallel domestication of tea trees.

We applied a Self-Organizing Map (SOM) approach (Wirth et al., 2011) to investigate the genetic diversity and the subpopulation-level distributions of characteristic alleles in tea cultivars. A gallery of SNP portraits helped visualize the genotypes (**Supplementary Figures S13-S15**) using color-coded allelic landscapes to depict major homozygous (blue), heterozygous (green), and minor homozygous (red), respectively. The accession portraits were very similar among accessions from the same geographic region but progressively different among accessions from different regions with increasing geographic distance. This result revealed three, two, and six smaller clusters in the CSA 1, CSA 2, and CSS groups, respectively. The geographic distribution of the CSA 1 group was mainly in Yunnan and Southeast Asia (**Supplementary Figure S16A**), whereas the CSA 2 group was in Africa, Bangladesh, Nepal, and Assam. By contrast, the CSS clusters were scattered across South China (**Supplementary Figure S16B**).

The population differentiation (*F_ST_*) level was more prominent between the CSA 1 and the CSA 2 clusters (**Supplementary Figure S17A**), indicating the genetic separation between Yunnan and South Asia tea tree plants. The *F_ST_* among CSS clusters was relatively lower (*F_ST_* < 0.15), showing a low level of genetic differentiation (**Supplementary Figure S17B**). Most CSS clusters showed higher nucleotide diversity (**Supplementary Figure S18A**), probably reflecting historical introgression and a more extensive habitat throughout evolution history in South China. The differences were significant between/among most CSA and CSS clusters (**Supplementary Figure S18B**).

To illustrate the high level of gene flow between the CSA and CSS groups, we estimated admixture graphs of SOM-defined clusters using TreeMix. The optimal number of migration events was four (**Figure 4B**, **Supplementary Figure S19**), including events from CSA 2 into CSA 1 (clusters 2 and 3; migration weight 0.393) and from CSS to CSA 1 (cluster 3; migration weight 0.340). This result suggested that CSA 1 cluster 3 might result from CSS and CSA 2 hybridization. We also observed an admixture event from CSS cluster 1 to CSA 2 cluster 1 (migration weight 0.154) and a gene flow from CSA 2 to CSS cluster 4 (migration weight 0.132).

### 3.6 *Camellia* pan-genome construction and analysis

**Figure 5:**
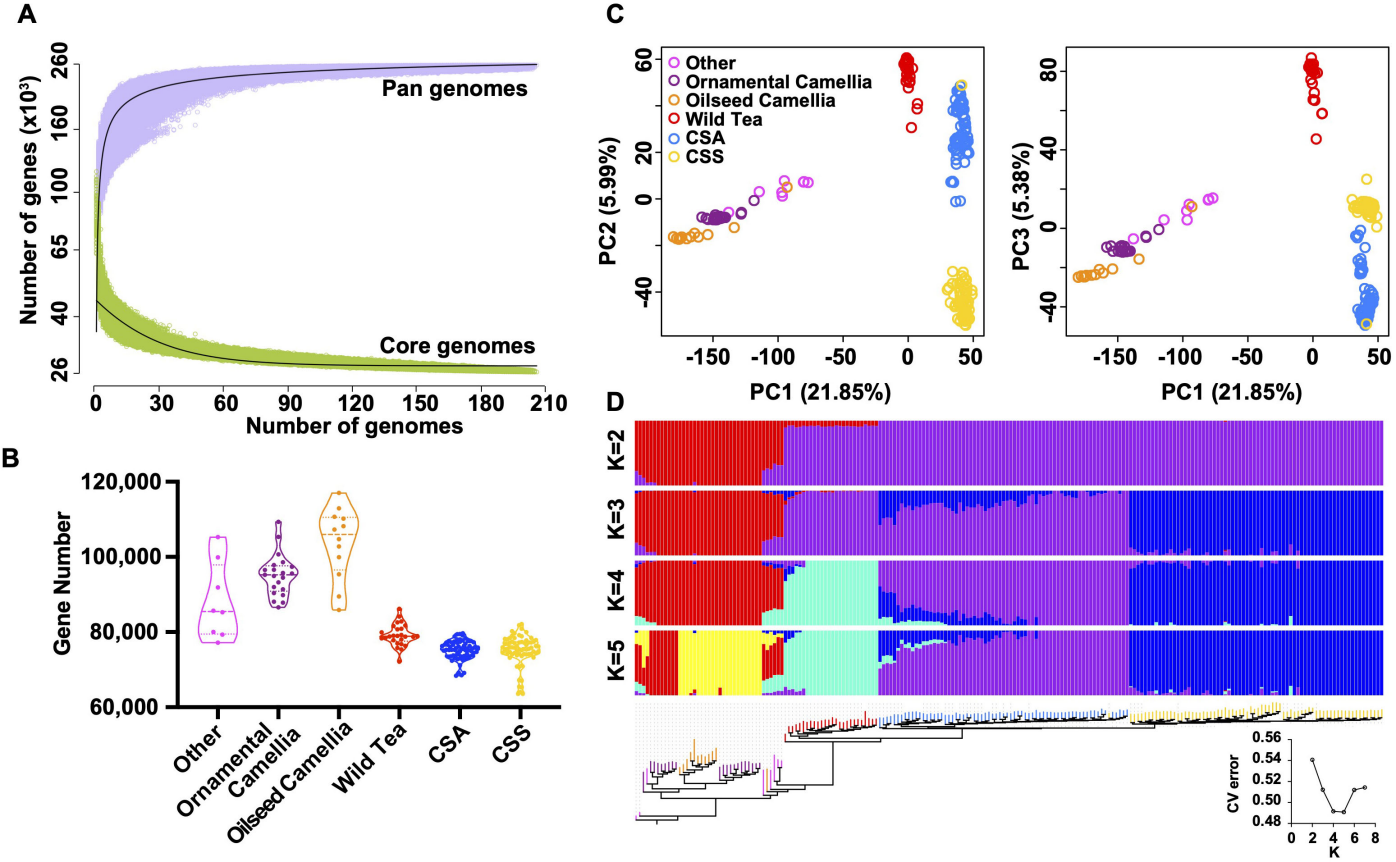
Feature of tea pan-genome. **(A)** Simulations of the increase of the pan-genome size and the decrease of core-genome size. Accessions were sampled as 10,000 random combinations of each given number of accessions. The upper and lower edges of the purple and green areas correspond to the maximum and minimum numbers of genes, respectively. Solid black lines indicate the pan- and core-genome curves fitted using points from all random combinations. **(B)** Violin plots showing the number of detected genes in each group. Three lines (from the bottom to the top) in each violin plot show the location of the lower quartile, the median, and the upper quartile, respectively. **(C)** Principal component analysis based on PAVs. The core and all absent genes were excluded. **(D)** Maximum-likelihood tree and model-based clustering of the 206 accessions with different numbers of ancestral kinships (K = 2, 3, 4, and 5) using the identified PAVs. The core and all absent genes were excluded.

We selected 206 *Camellia* accessions (**Supplementary Table S16**) for the pan-genome construction, including eight accessions in the Other *Camellia*, 21 accessions in the ornamental *Camellia*, 12 accessions in the oilseed *Camellia*, 26 accessions in the Wild Tea group, 68 CSA accessions, and 71 CSS accessions. The average genome size of accessions was ~2.30 Gb for the *de novo* assemblies, and the average N50 size was 1.46 kb (**Supplementary Figure S20**). We mapped assembled contigs to the ‘YK10’ reference genome and identified ~43.33 Gb non-reference sequence sharing < 86% identity with the reference (**Supplementary Figure S21**). After removing redundancies, we obtained 9,275,358 sequences (8.58 Gb) representing the non-reference genome and annotated 216,302 protein-coding genes. The ‘YK10’ reference and the final non-reference genome formed the *Camellia* pan-genome and were used for the following analyses. We categorized pan-gnome genes according to their presence frequencies, including 26,330 core genes shared by all 206 accessions (100% accessions), 4,497 softcore genes (99-100% accessions), 206,044 shell genes (1-99%), and 21,916 cloud genes (<1%), respectively (**Supplementary Figure S22**). Iteratively random sampling of accessions showed the plateau of both pan and core genes, suggesting a finite pan-genome size (**Figure 5A**).

The tea tree *Camellia* accessions have significantly fewer genes than the Other *Camellia* species (**Figure 5B**, **Supplementary Figure S23**), suggesting a general trend of gene loss during tea tree evolution. Phylogenetic, principal component analyses, and ADMIXTRUE clustering (**Figures 5C, D**, **Supplementary Figure S24**) using the presence/absence variations (PAVs) showed clear separation of the Wild Tea, CSA, and CSS accessions except for re255, which may be a misidentified accession. By contrast, most Other *Camellia* accessions were clustered together, corresponding to the results from similar analyses using SNPs (**Supplementary Figure S7**).

We identified PAVs under selection by screening genes with significantly different frequencies between CSA and CSS (**Supplementary Figure S25**). We identified 11,060 CSA-favorable genes (higher frequencies in CSA) and 10,972 CSS-favorable genes (higher frequencies in CSS). The CSA-favorable genes were mainly involved in carotenoid biosynthesis, metabolism of terpenoids and polyketides, carbohydrate metabolism, and benzoxazinoid biosynthesis (**Supplementary Table S21**). The CSS-favorable genes were mainly involved in carotenoid biosynthesis, steroid biosynthesis, metabolism of terpenoids and polyketides, cytochrome P450, nitrogen metabolism, and monoterpenoid biosynthesis (**Supplementary Table S21**). Similarly, we identified 210 ornamental *Camellia*-favorable genes and 2,945 oilseed *Camellia*-favorable genes (**Supplementary Figure S26**). The pathways of fatty acid degradation and carbohydrate metabolism were enriched in oilseed *Camellia*, but no significant pathways related to secondary metabolism were enriched in ornamental *Camellia*.

## 4 Discussion

Tea is a popular non-alcoholic beverage worldwide. Because of the health benefit, economic value, and progress of sequencing technique, lots of *Camellia* genomes, including ‘YK10’, CSA, CSS, and oilseed *Camellia*, have been decoded (Xia et al., 2017; Wei et al., 2018; Wang et al., 2020; Xia et al., 2020; Zhang et al., 2020a, 2020, 2021; Lin et al., 2022; Chen et al., 2023). However, most CSA assemblies have lower contig N50 lengths (~881 kb) (Chen et al., 2023). Our new ‘YK10’ assembly has improved forty-fold in terms of contig N50 length (817 kb) compared to a previous report (Xia et al., 2017), and its quality was comparable with other reported CSA (Chen et al., 2023) and CSS genomes (Wei et al., 2018; Wang et al., 2020; Xia et al., 2020; Zhang et al., 2020a). TE burst events were estimated at around 0.331 MYA using our genome, which was younger than that obtained from a previous ‘YK10’ genome (Xia et al., 2017). Furthermore, the Ks peak of WGD analysis in this study (0.386-0.414) was larger than that in the previous studies (0.31-0.36) (Wang et al., 2020; Xia et al., 2020), suggesting an older genome duplication event. Observably, our results showed that some *NBS-LRR* and phenylpropanoid biosynthesis-related gene families were expanded in the ‘YK10’ genome, which may be beneficial for disease resistance and tea flavor, respectively, given the fact that *NBS-LRR* genes are the most important disease resistance genes in plants (Wu et al., 2017). The cardiovascular health benefits of drinking tea are mainly due to flavonoids (Hodgson and Croft, 2010), which are synthesized through a specific branch of the phenylpropanoid pathway (Li et al., 2019).

Trait diversification of *Camellia* domesticates was visualized for three main types according to human needs, but the potential genomic drivers remained covered. Ornamental *Camellia* cultivars exhibit diverse flowering times, flower sizes, petal colors, and petal forms. These traits are putative targets of human selection throughout history. We identified abundant selective sweep genes related to floral development in this group, among which the ARF protein plays a role in the auxin-mediated pathway in promoting floral fate (Chung et al., 2019) in addition to its role in regulating gynoecium morphogenesis, self-incompatibility, *de novo* organ regeneration, and organ polarity (Zhang et al., 2018). MYB proteins regulate anthocyanin biosynthesis in fruits and flowers, and GST proteins transport anthocyanin from the endoplasmic reticulum to facilitate the coloration of flowers, leaves and stems (Lu et al., 2021). Other selected genes in the group encoding beta-ring hydroxylase protein and ethylene-responsive transcription factor protein also affected flowering in *Camellia* (Wang et al., 2016; Huang et al., 2022), thus possibly influencing the ornamental value of flowers in ornamental *Camellia*.

The oilseed *Camellia* produces high-temperature cooking oil with a mild tea aroma. It is a crucial source of dietary fats for the local Southeast Asian people. Not surprisingly, some selective sweep genes in this group were found to be related to FA biosynthesis. In *Camellia*, phospholipase D (PLD) became important in regulating triacylglycerol (TAG) production. Transgenic *Camelina* plants expressing *Arabidopsis* PLD produced 2-3% higher levels of TAG (Deepika and Singh, 2022). In addition, acyl-CoA synthetase mediates *de novo* lipogenesis and glycerol lipid assembly in developing seeds (Ichihara et al., 2003). Furthermore, the genes encoding fatty acid omega-hydroxylase proteins and fatty acyl-ACP thioesterase B protein are also related to FA metabolism (Salas and Ohlrogge, 2002; Osborne et al., 2022).

The details of tea tree domestication remain inconclusive, as the true progenitor of the cultivated tea tree is unknown. New evidence emerged to propose the parallel cultivation of CSA and CSS in the past and hypothesize that selected genes possibly affected flavor, leaf size, and tree height (Zhang et al., 2021). Our results revealed that the function enrichment of selective sweep genes showed differences in these two groups. We also found that CSA and CSS were sister clades in the phylogenetic tree, and the divergence time between CSA and CSS predated the earliest documentation of tea cultivation. Ecological niche models showed that the suitable habitat of tea plants gradually separated into two smaller regions since the Late Holocene. All of these supported the parallel domestication processes for CSA and CSS. However, the location and time of domestication still require verified progenitor populations for elucidation. Especially, a previous study found that both cell length and the cell number of internodes were reduced in a dwarf *mini plant 1 (mnp1)* mutant of *Medicago truncatula*, which involved a putative CPS gene in the first step of gibberellin biosynthesis (Guo et al., 2020). A CPS gene was found in the selective sweep region in the CSS group in the present study, providing indirect evidence for the relatively shorter plant height in CSS.

The kinship of important cultivars contributed to understanding the history of breeding and screening germplasm resources, e.g., identifying the offspring of the cultivar ‘Yabukita’. The pattern of geographic distribution among different CSA and CSS subpopulations showed significant differences, suggesting that established cultivars traveled to far-away tea-growing regions, possibly with early farmers. In addition, we investigated the population differentiation, nucleotide diversity, and gene flow among these subpopulations. These results reflected a complex hybridization history during tea tree domestication and breeding history.

Furthermore, we provided a landscape of *Camellia* pan-genome. Though a graphical pangenome based on the *de novo* assembly of 22 elite tea cultivars has been released (Chen et al., 2023), our *Camellia* pan-genome will expand the *Camellia* gene pool, thus helping subsequent functional research. Genetic structure analysis based on PAVs of *Camellia* pan-genome corresponded to the results based on SNPs, proving the capacity and value of this genetic variation. The group-favorable genes also provided valuable genetic resources for explaining the trait diversification of *Camellia* domesticates.

## 5 Conclusion

In conclusion, we presented a high-quality genome sequence for the CSA tea tree. According to resequencing and pan-genome analyses for *Camellia* species, the genetic diversity and population structure of a worldwide panel were revealed, and genetic drivers of selection during domestication were identified. Our results offered a valuable genomic resource for ongoing functional research of *Camellia* species in agriculture. Many aspects of the tea origin remain unknown, but our results agreed with the parallel domestication of CSA and CSS. Because the progenitor of tea trees is unidentified, two-population analyses of selective sweeps and the dispersal history of tea trees remained to be examined.

## Data Availability

The raw reads of genome sequencing data are available at the China National Center for Bioinformation under the accession number PRJCA017951. The raw reads of resequencing data are available at the China National Center for Bioinformation under the accession number PRJCA017952. Genome assembly and annotation are available at Teabase (http://teabase.ynau.edu.cn).
