# Supplementary material for: Genomic analyses of agronomic traits in tea plants and related Camellia species
Source: Front Plant Sci. 2024 Aug 26;15:1449006. doi: 10.3389/fpls.2024.1449006 (PMC11381259; doi:10.3389/fpls.2024.1449006)
Supplement: Supplementary file 1 [file Table1.docx]

**Supplementary Figures**


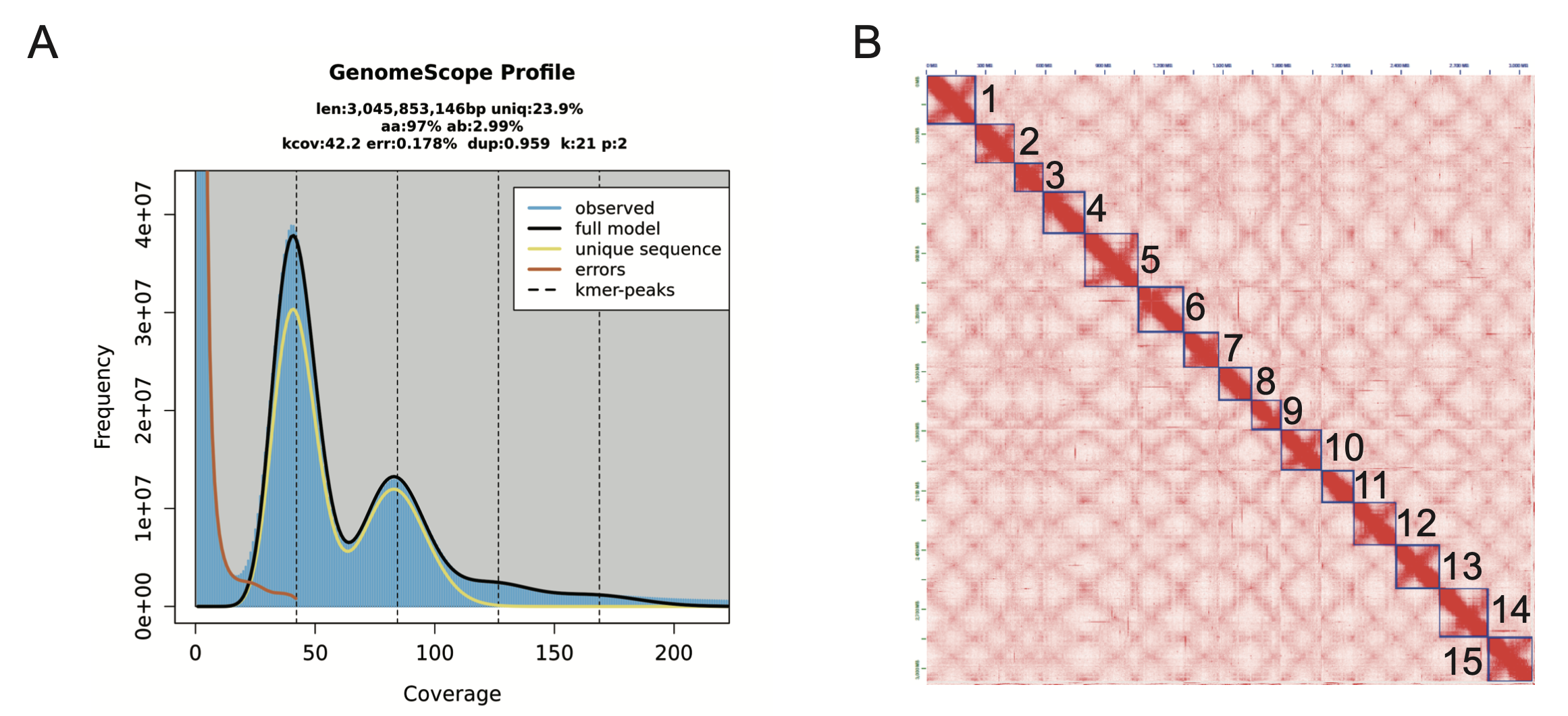


Figure S1.

**Genome survey and** **Hi-C interactions.** (A) Genome survey using k-mer distribution of sequencing reads. The K-mer distribution were constructed basing on 21-mer. (B) Heatmap of Hi-C interactions for ‘YK10’ chromosomes (Chr1-Chr15).

Supplementary Figures


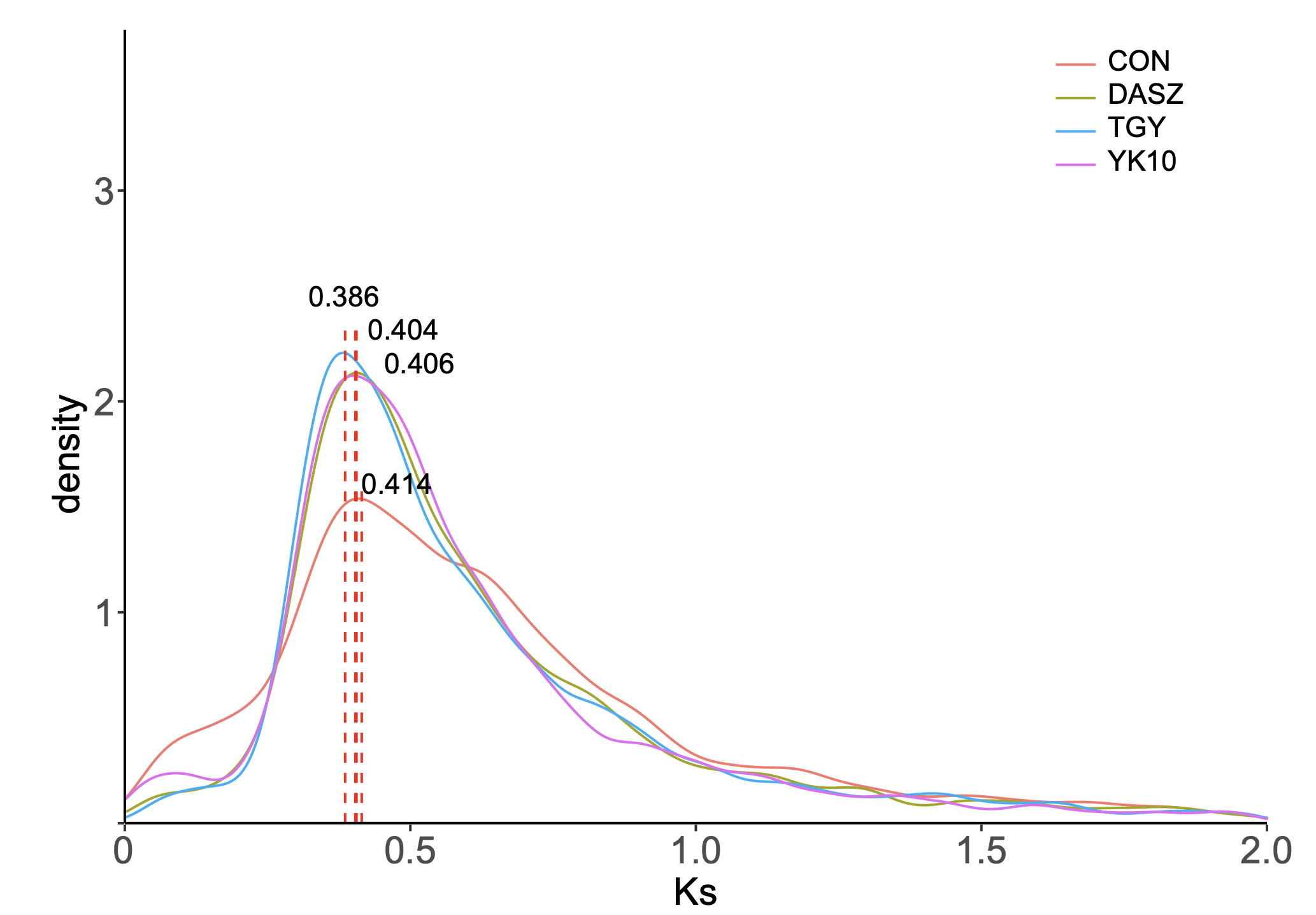


Figure S2.

**The Ks distribution of paralogous gene pairs in different species.** YK10: CSA cultivar ‘Yunkang10’ (this study); TGY: CSS Chinese Oolong tea variety ‘Tieguanyin’; CON: wild oilseed *Camellia* (*C. oleifera* var. ‘Nanyongensis’); DASZ: an ancient tea tree.


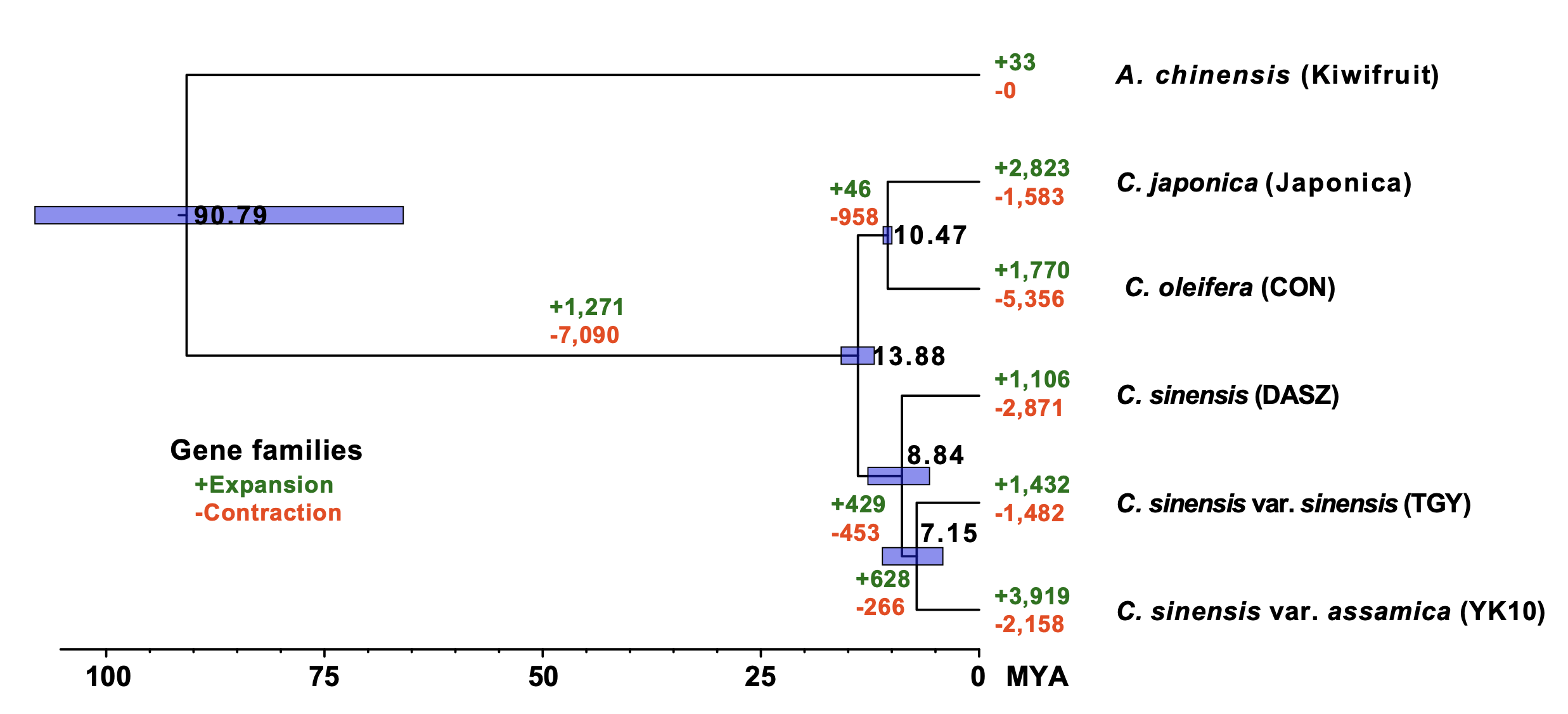


Figure S3.

**Expansion and contraction of gene families.** The number of green/red show the expanded/contracted gene families in each plant species.


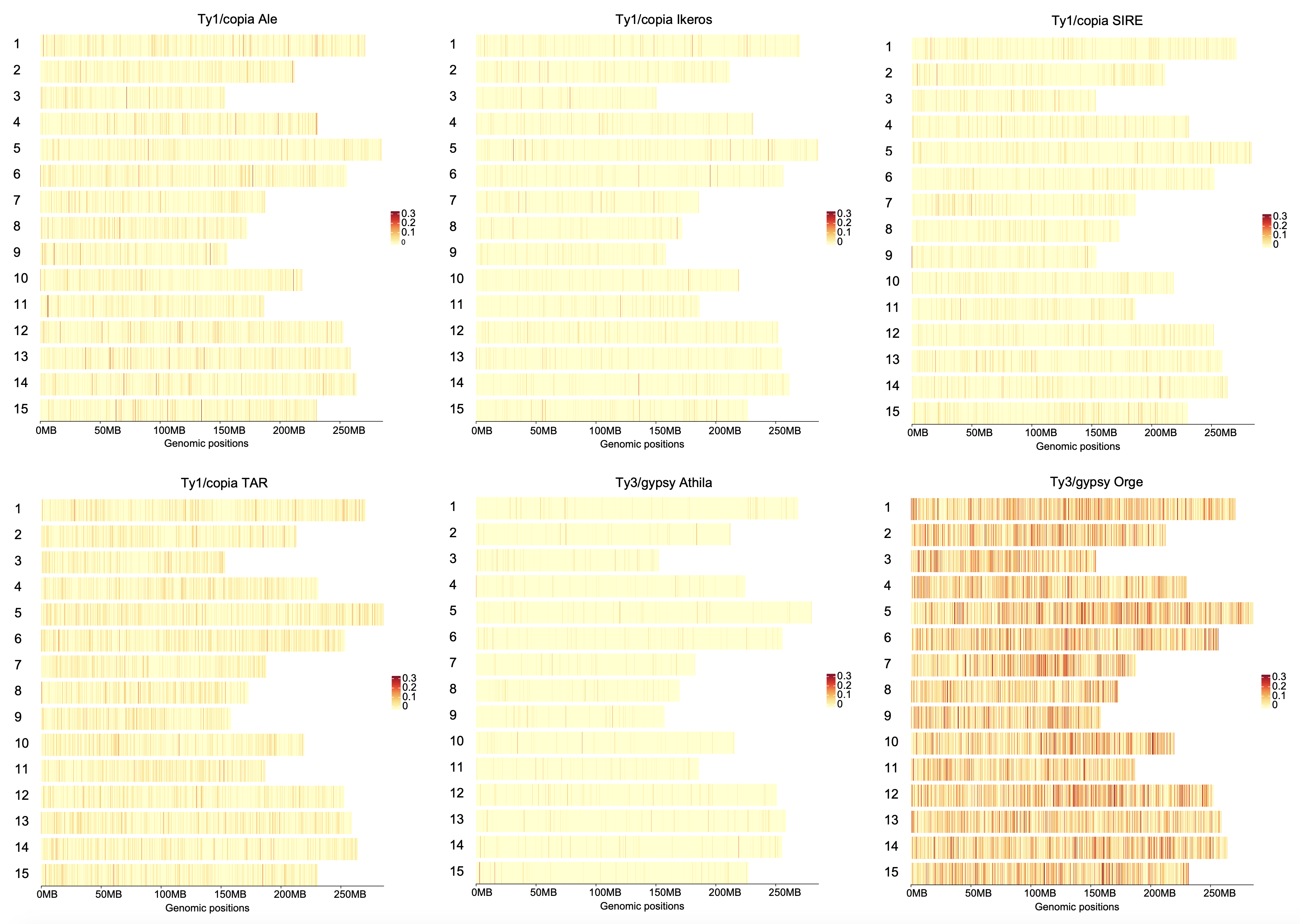


Figure S4.

**The density of different TE lineages inferred from the detection of their protein-coding domains along chromosome.**


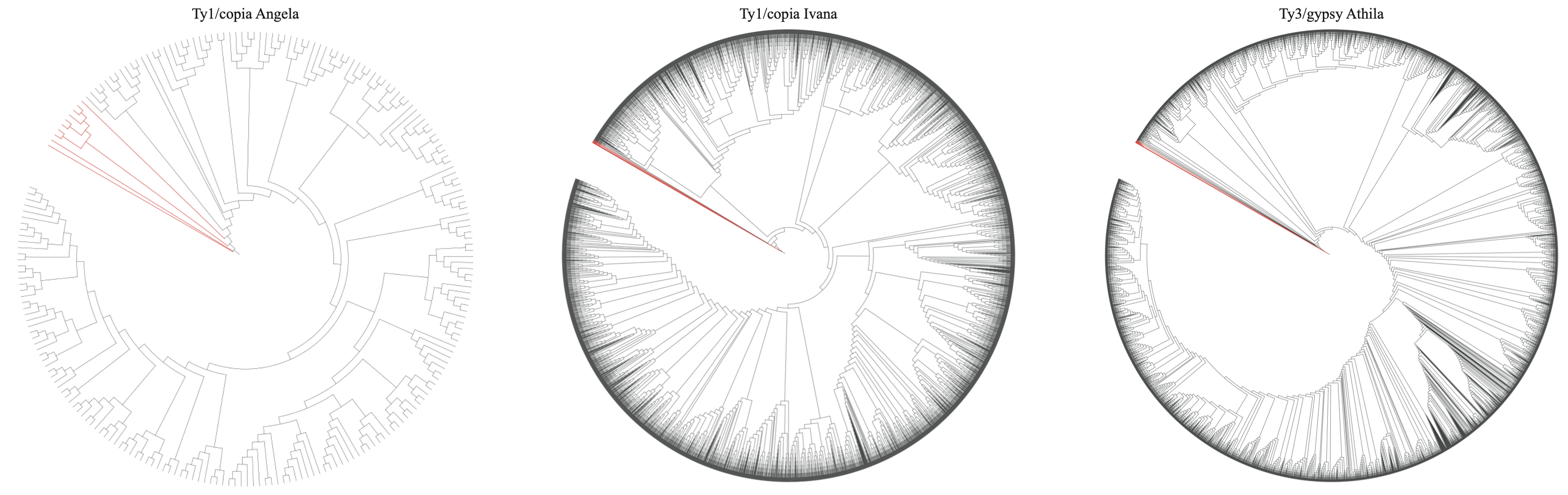


Figure S5.

**Phylogenetic trees of TEs.** The trees were built from RT domain sequence similarities among different lineage-specific copies identified in the ‘YK10’ genome. Deep branching revealed ancient expansion while flat branching is consistent with a recent burst of insertion activity. Origin branches (red) correspond to outgroup sequences.


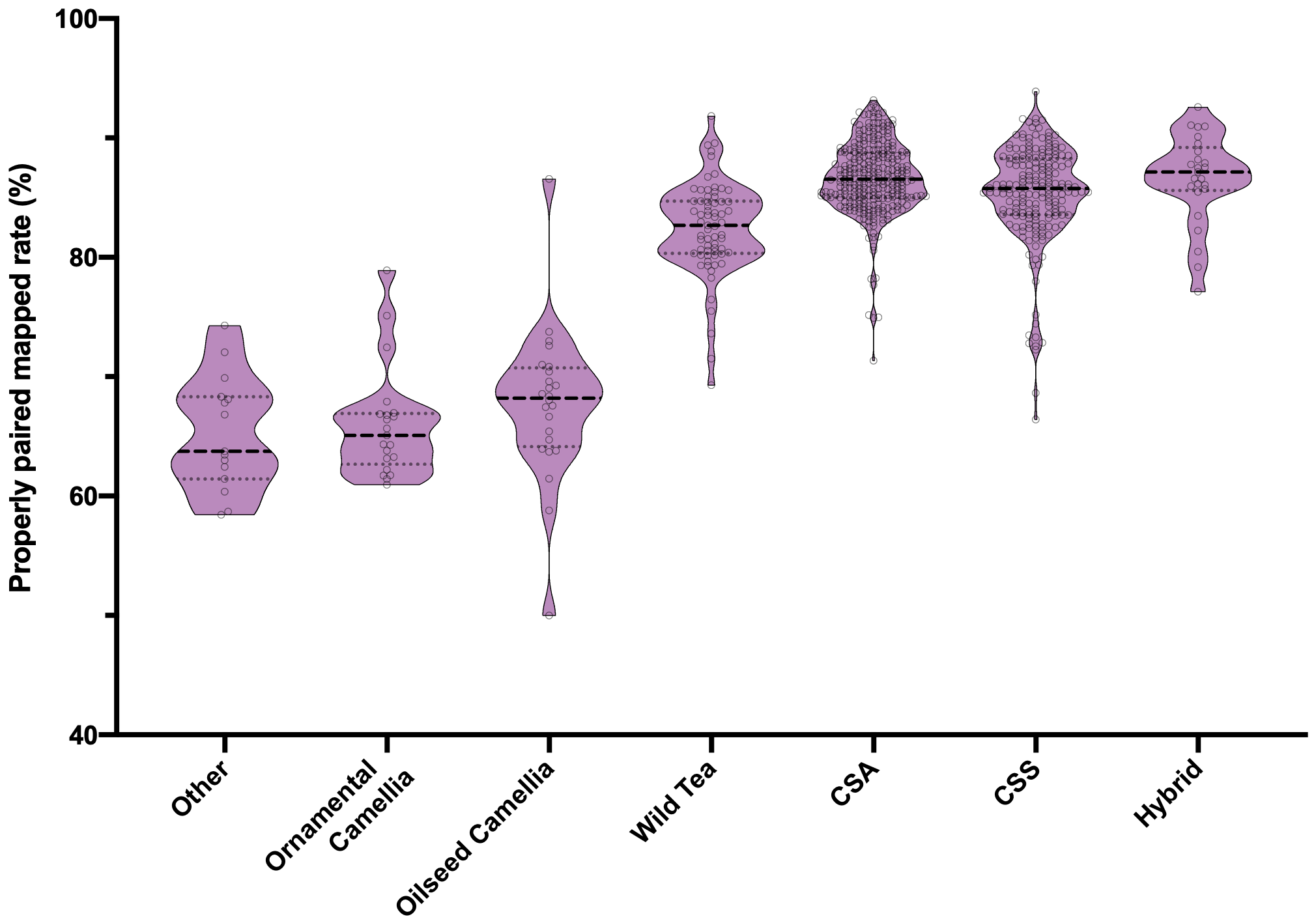


Figure S6.

**Violin plots showing the rate of properly paired mapped to YK10 genome in each group.** Three lines (from the bottom to the top) in each violin plot show the location of the lower quartile, the median and the upper quartile, respectively.


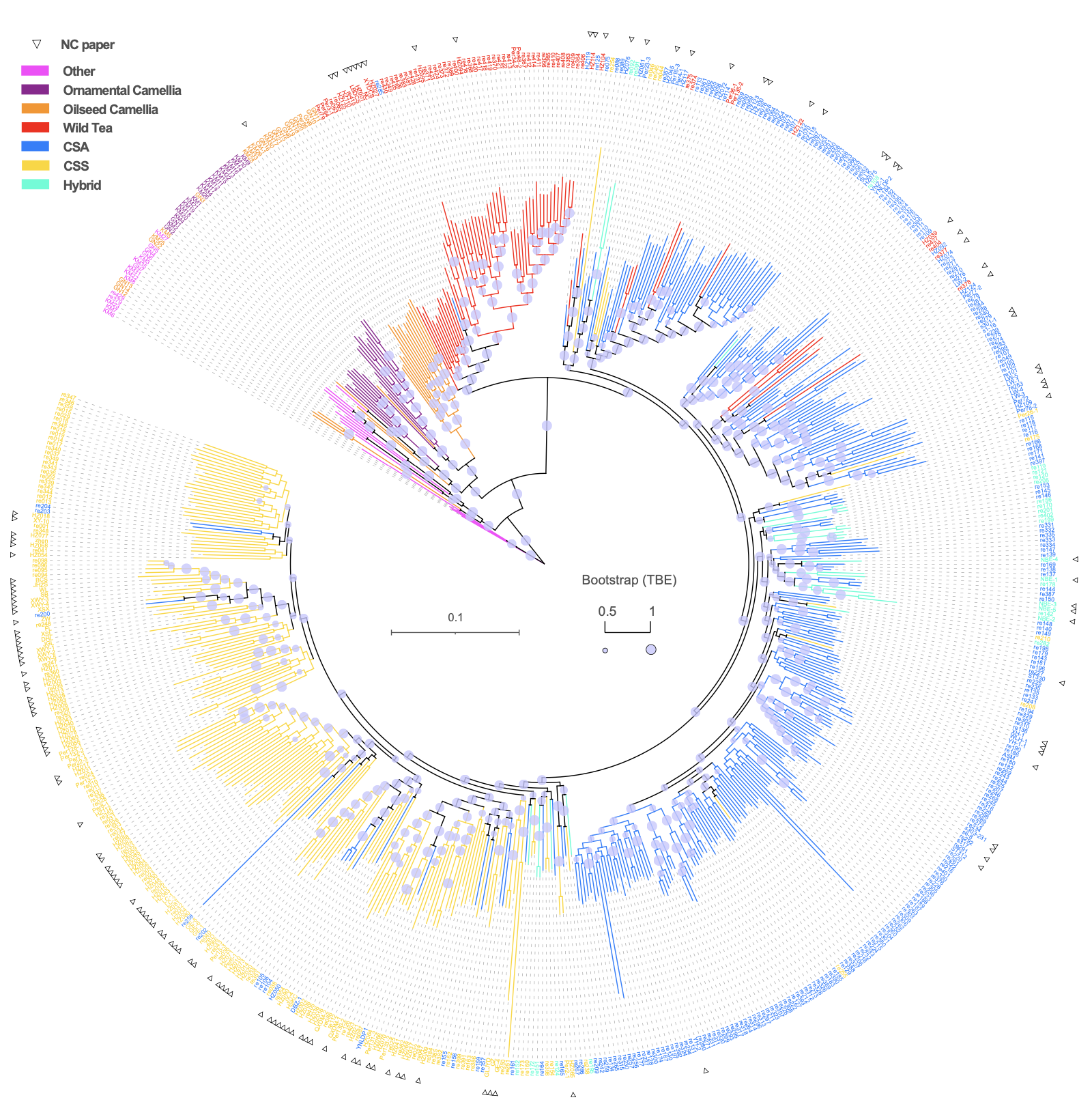


Figure S7.

**Phylogenetic tree of *Camellia* accessions.** Bootstrap values were indicated by blue circles.


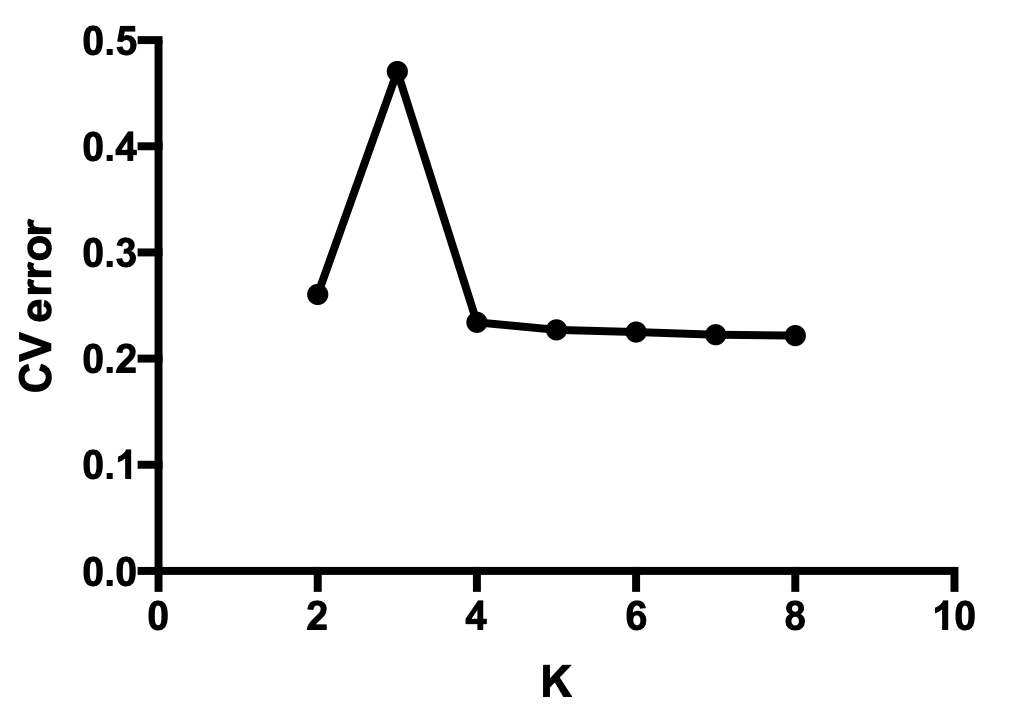


**Figure S8. Cross-validation error plot for the ADMIXTURE analysis.** Because the decrease of CV error is very slow from K=5, so we decided to select K=5 as the optimal value.


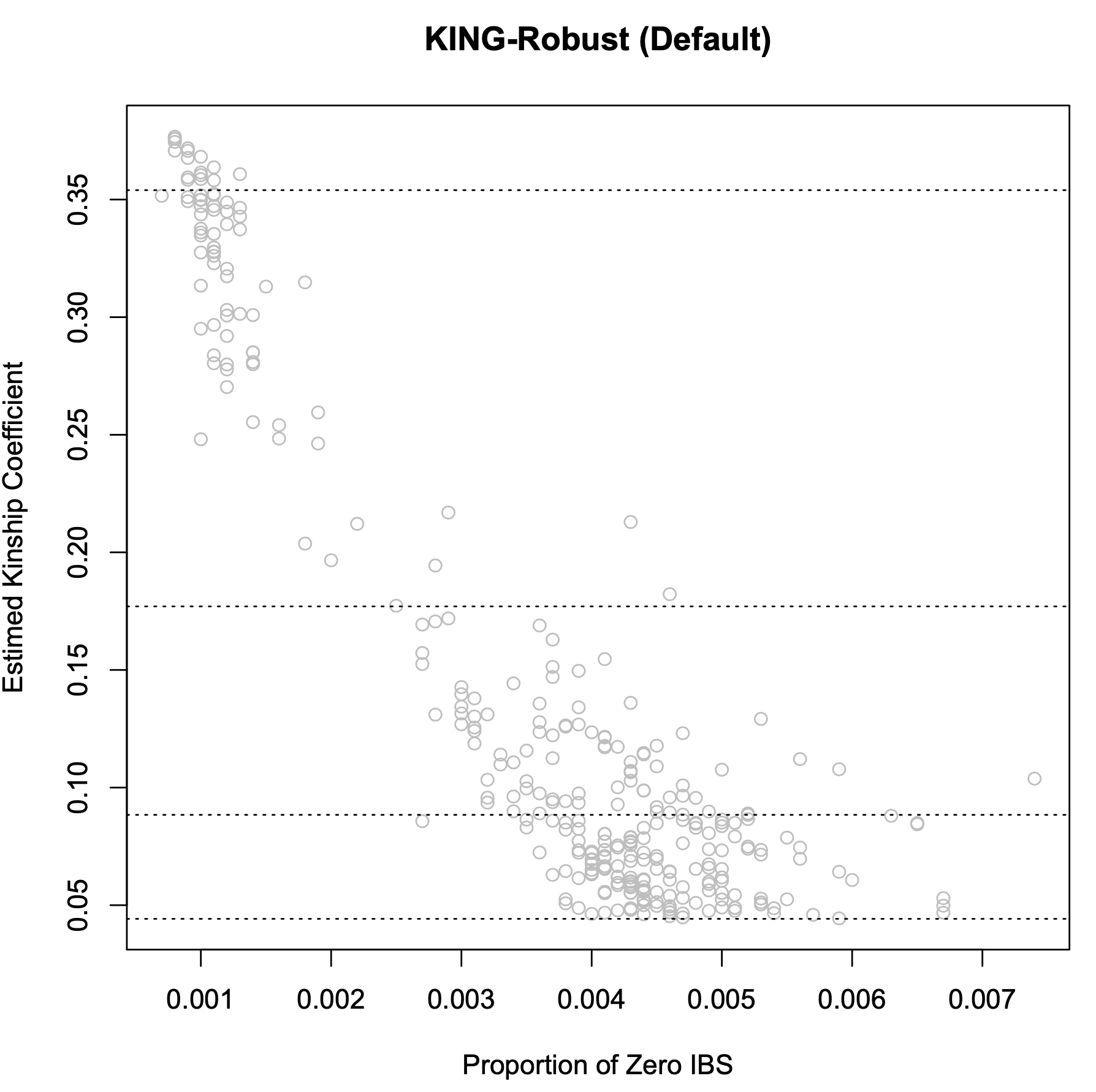


Figure S9.

**Relationship between each accession using estimated kinship coefficient.** The dash lines show the range >0.354, [0.177, 0.354], [0.0884, 0.177] and [0.0442, 0.0884] corresponds to duplicate, 1st-degree, 2nd-degree, and 3rd-degree relationships respectively.


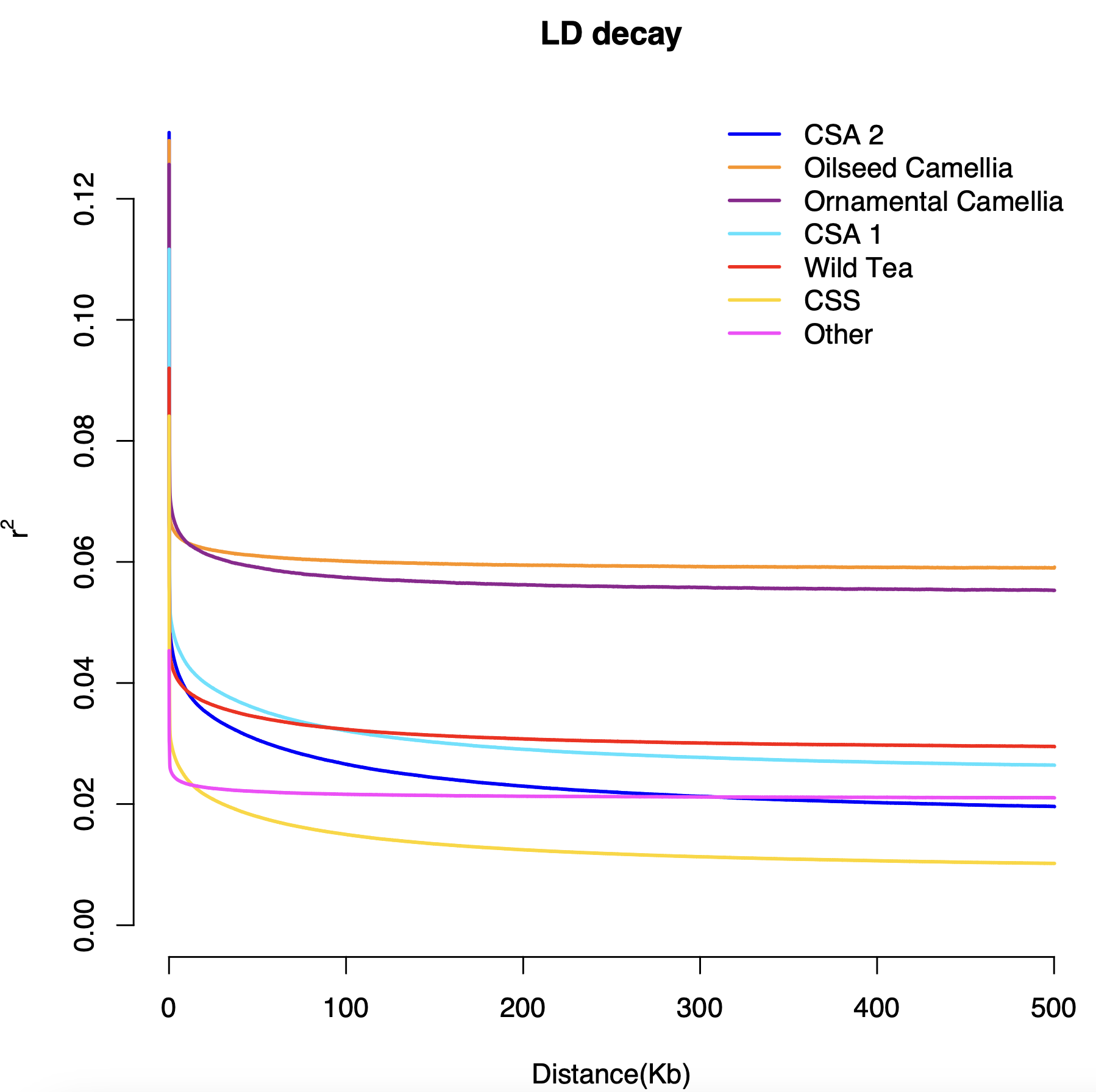


Figure S10.

**Decay of linkage disequilibrium in Other, Wild Tea, CSA 1, CSA 2, CSS, ornamental *Camellia* and oilseed *Camellia* group.**


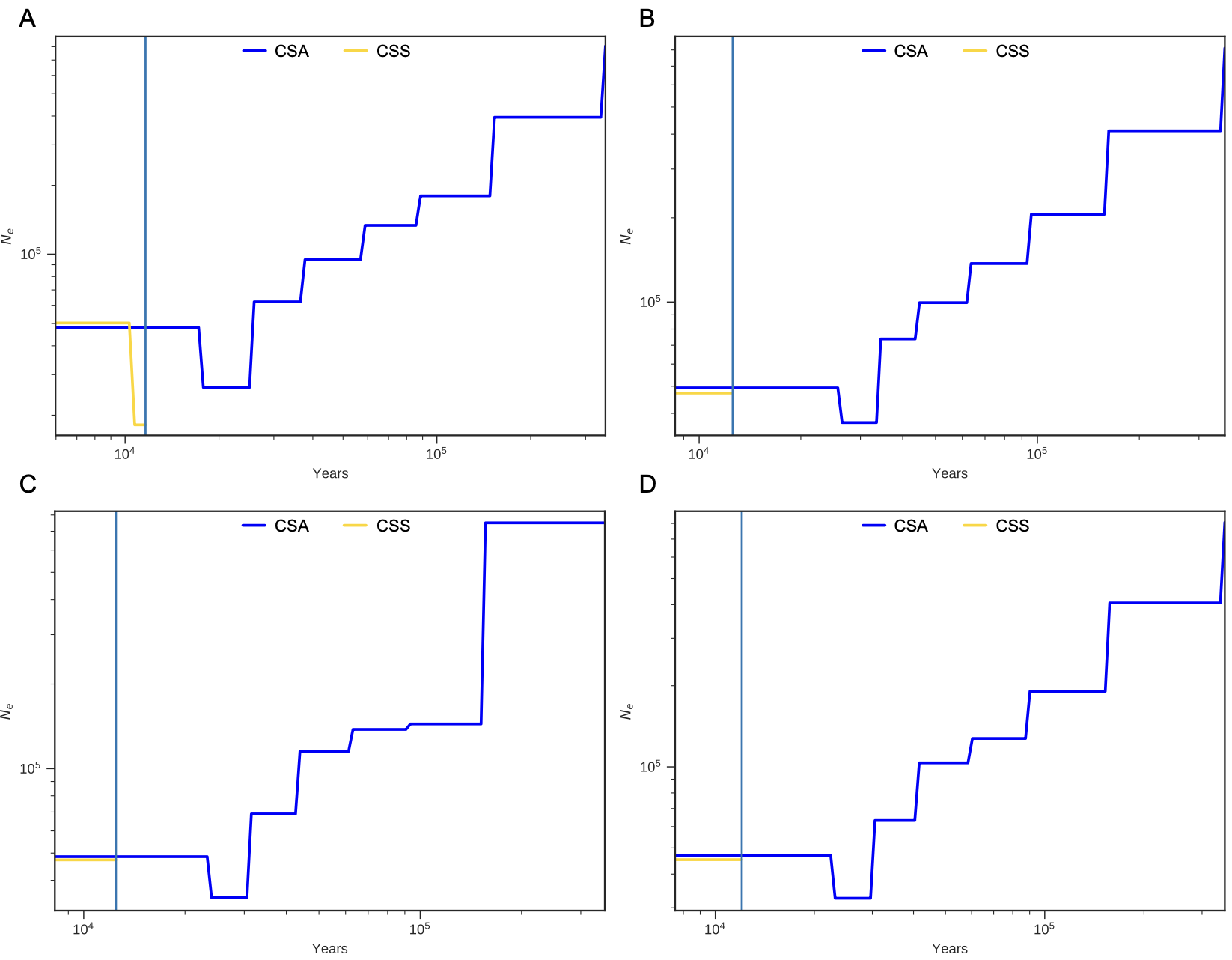


Figure S11.

**Demographic history of CSA 2 and CSS group.** The plots were deduced from SMC++ split analysis for no mask runs of homozygosity (A) and automatically treat runs of homozygosity longer than 50kb (B), 100kb (C), 200kb (D) base pairs as missing.


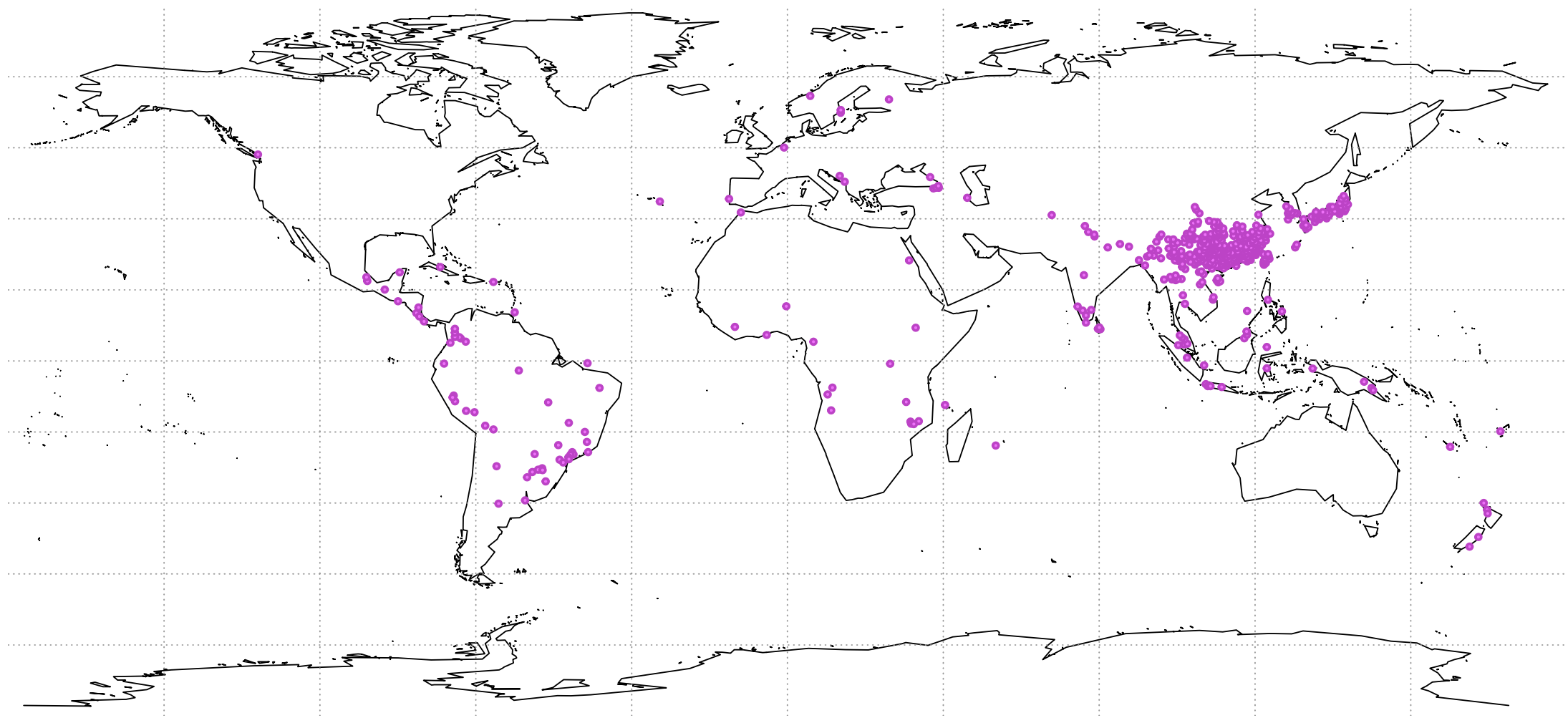


Figure S12.

**Geographical distribution of occurrence records for tea.**


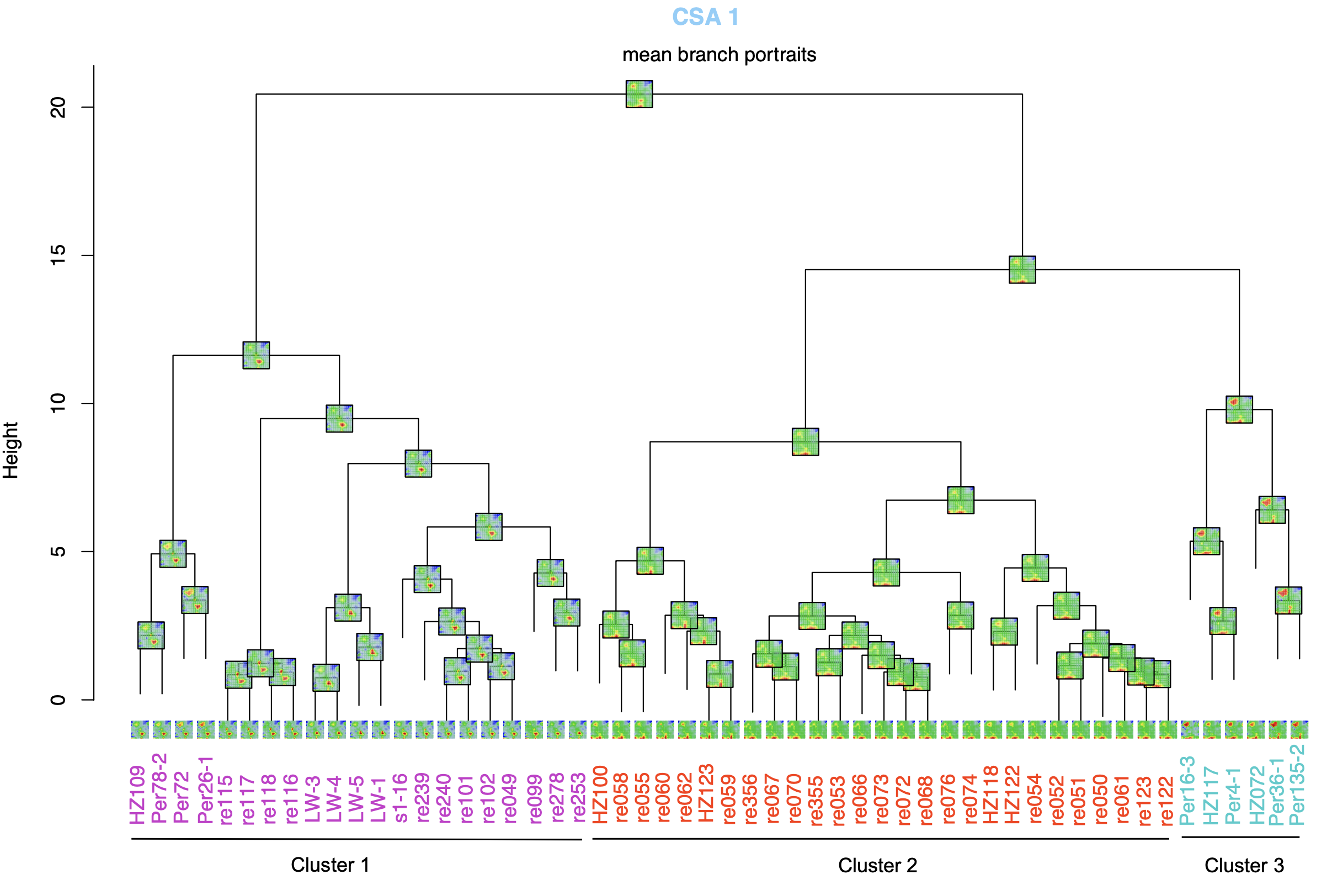


Figure S13.

**Clustering based on SNP portraits of CSA 1 group.**


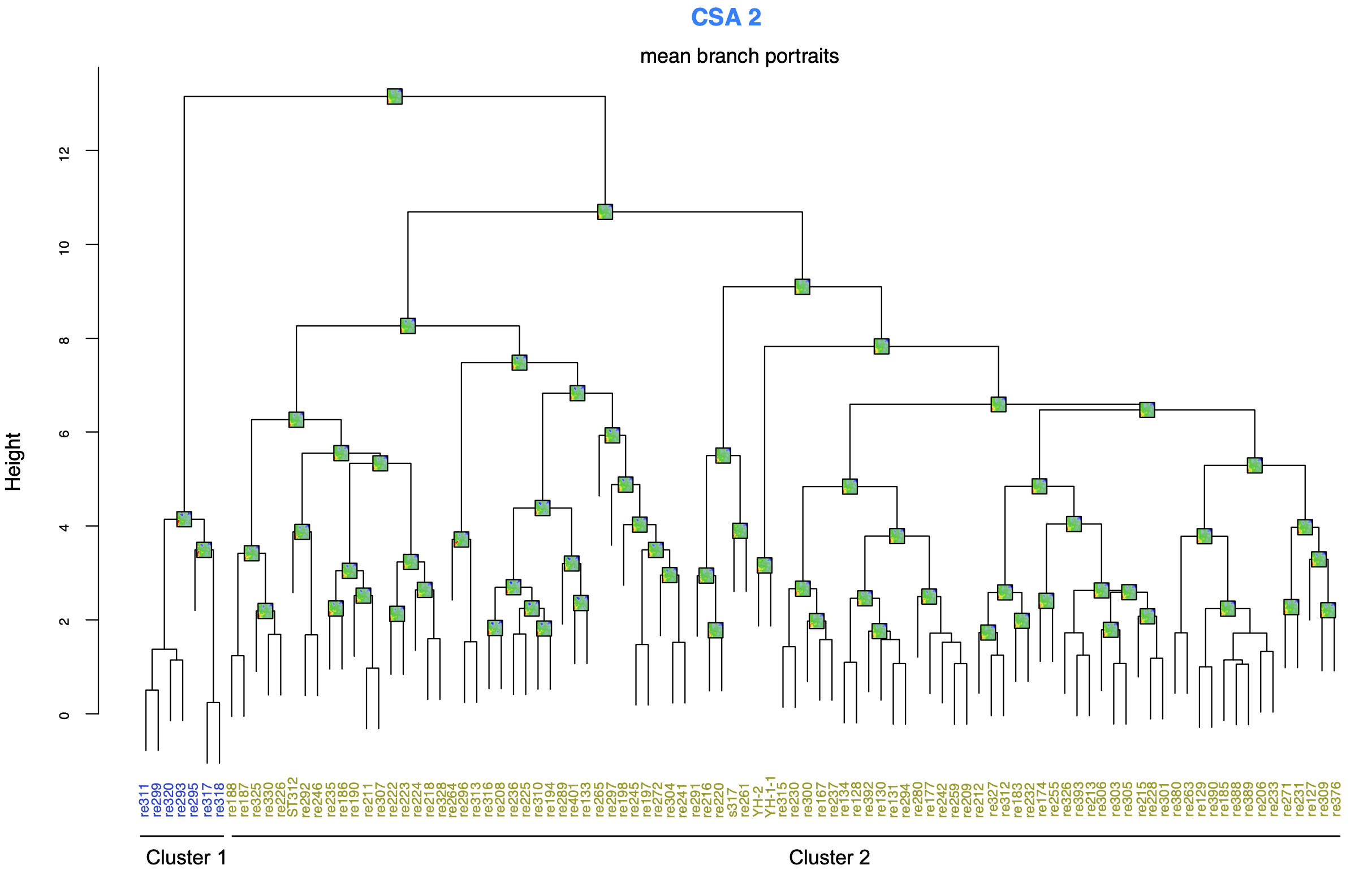


Figure S14.

**Clustering based on SNP portraits of CSA 2 group.**


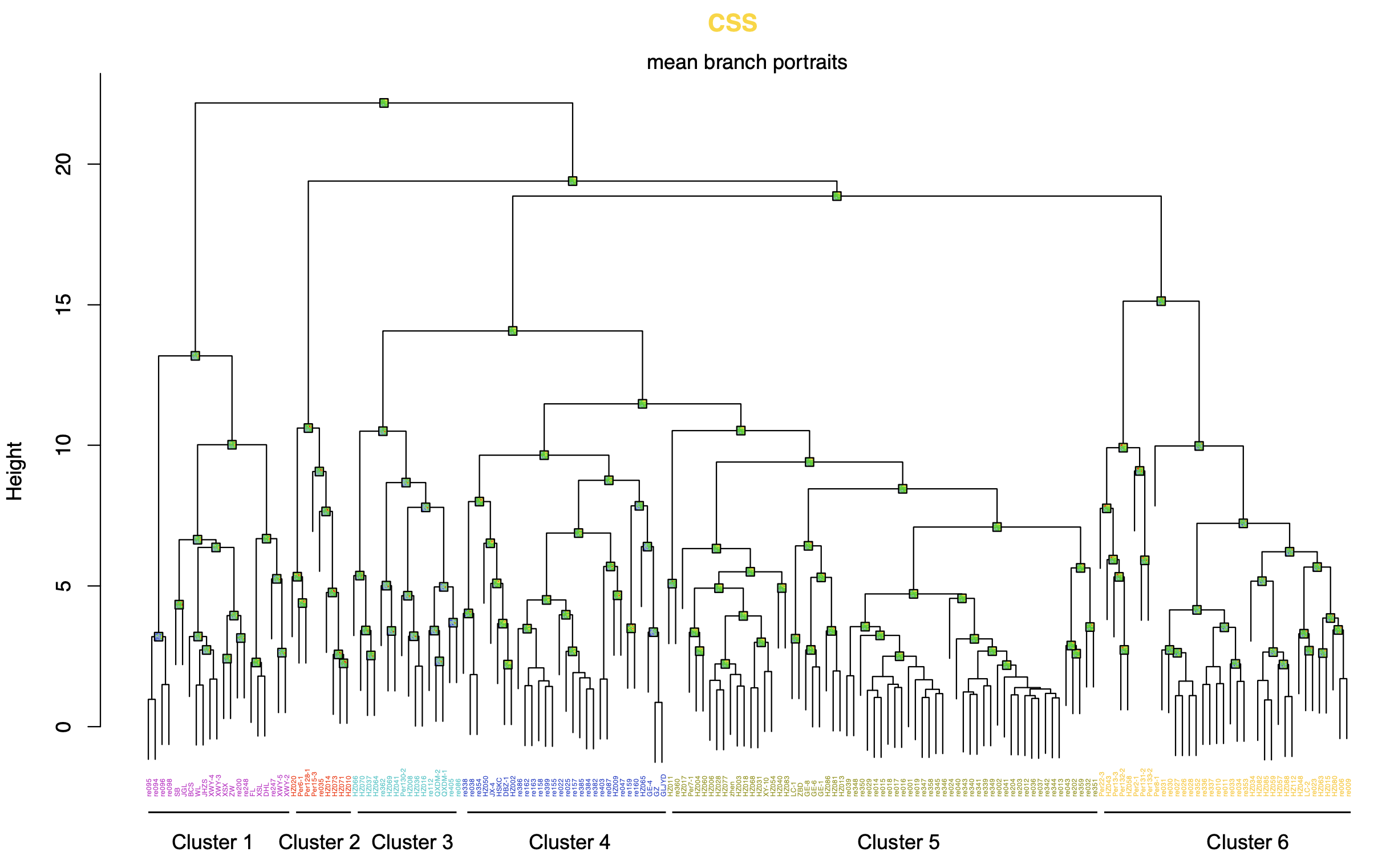


Figure S15.

**Clustering based on SNP portraits of CSS group.**


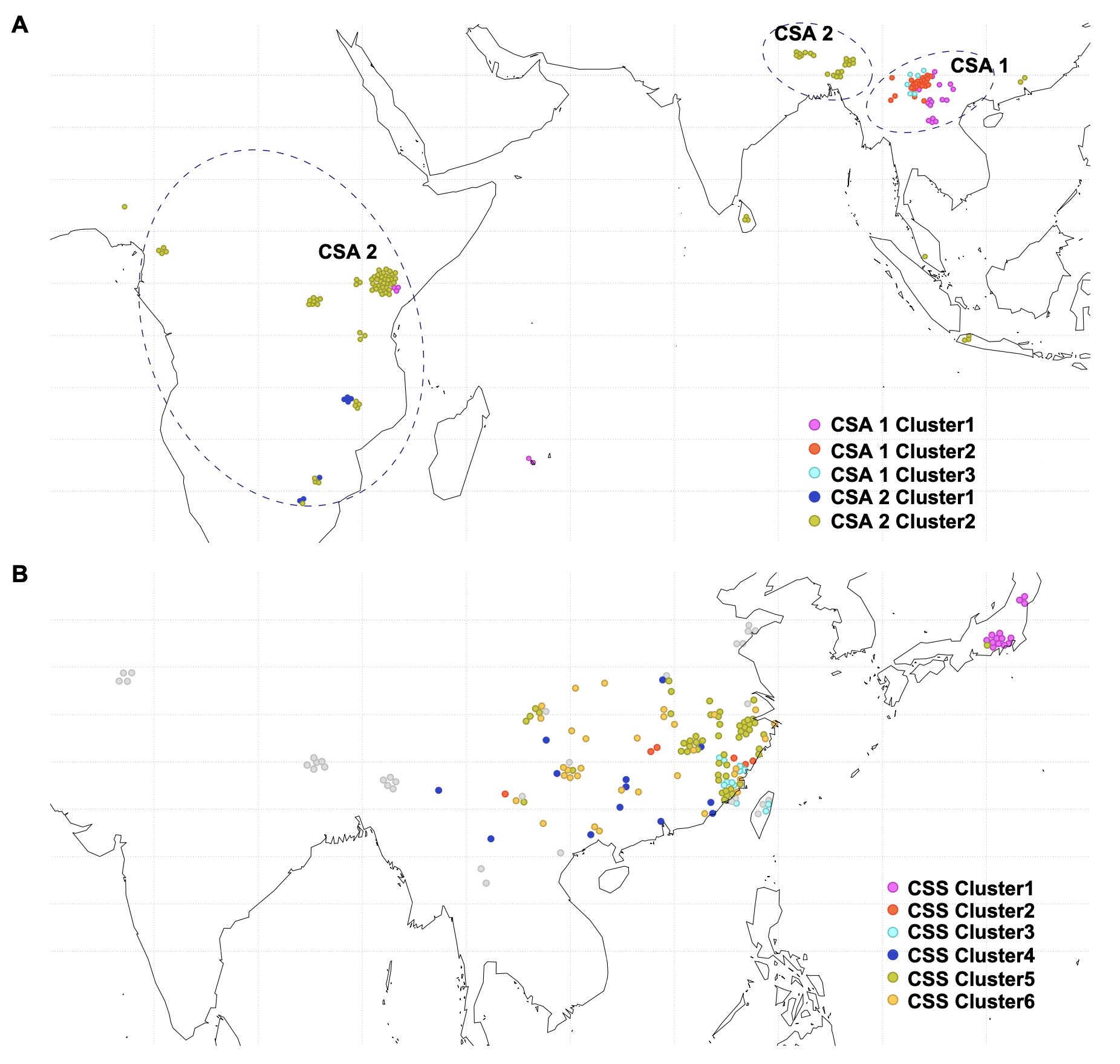


Figure S16.

**Geographical distribution of different CSA and CSS clusters.** (A) Geographical distribution of different CSA clusters. (B) Geographical distribution of different CSS clusters. Grey dots represent that the accessions were introduced.

**
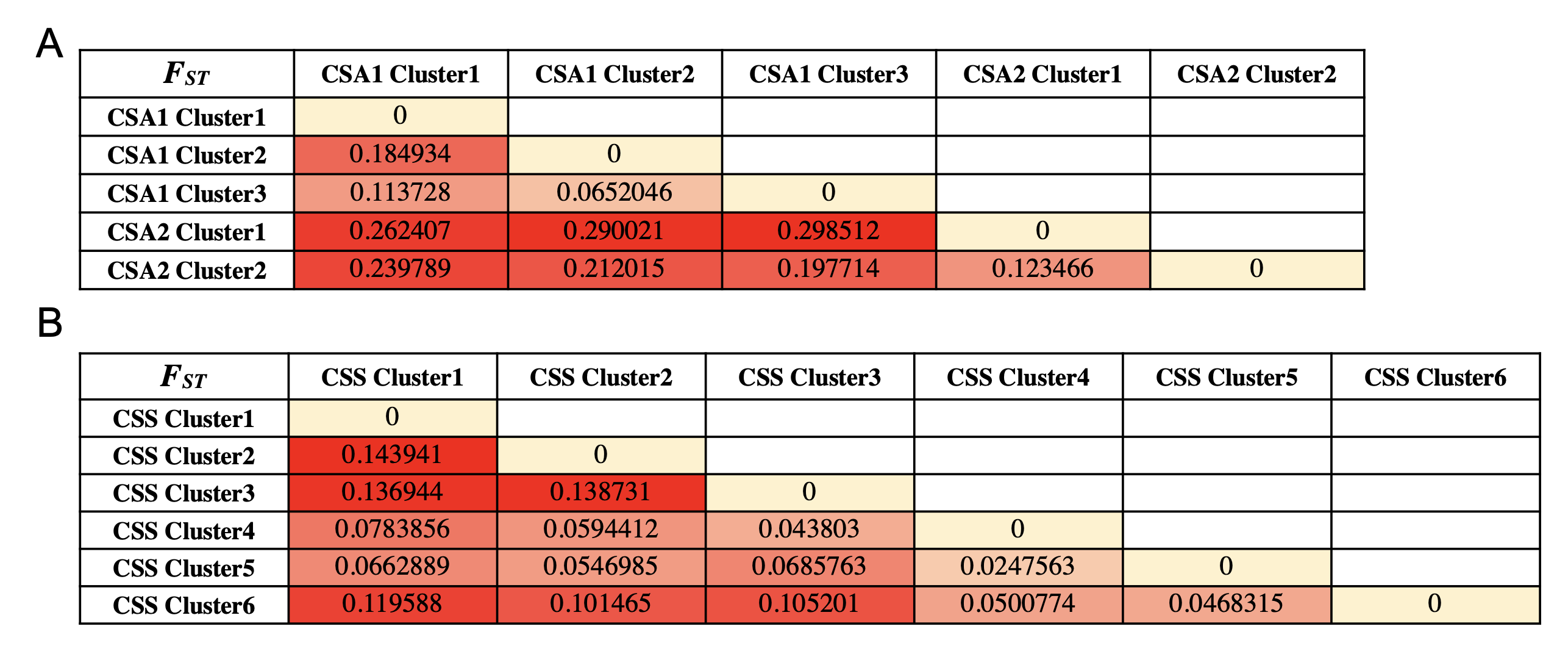
**

Figure S17.

**Heatmap of *F_ST_* among different clusters for CSA (A) and CSS (B) group.**


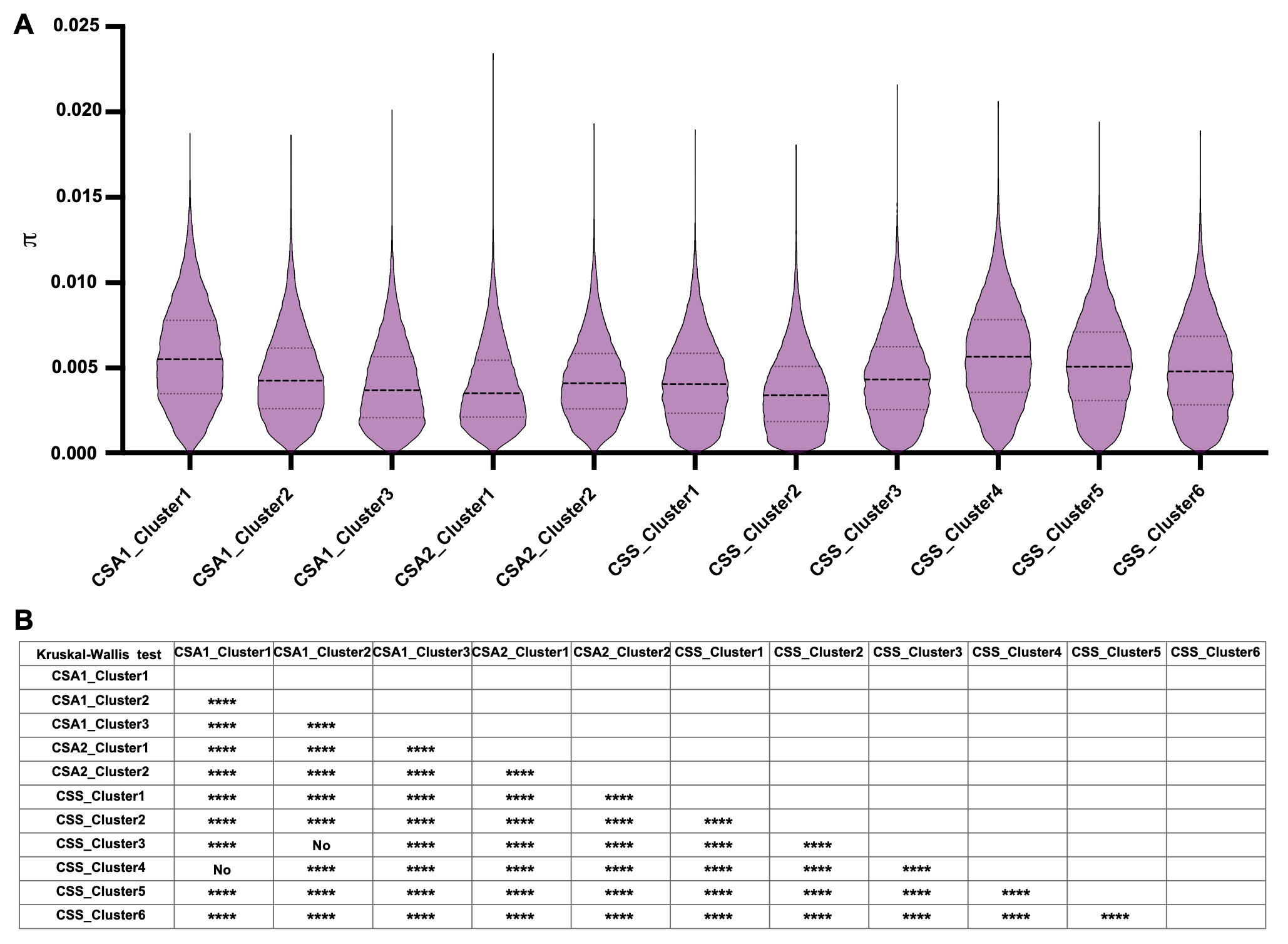


Figure S18.

**The distribution of π and significance analysis among different clusters.** (A) Violin plot show the distribution of π for different clusters in CSA and CSS group. Thick dash line and fine dash lines represent median and interquartile range. (B) Statistically significant difference between the different clusters in CSA and CSS groups using Kruskal-Wallis test (****P < 0.0001).


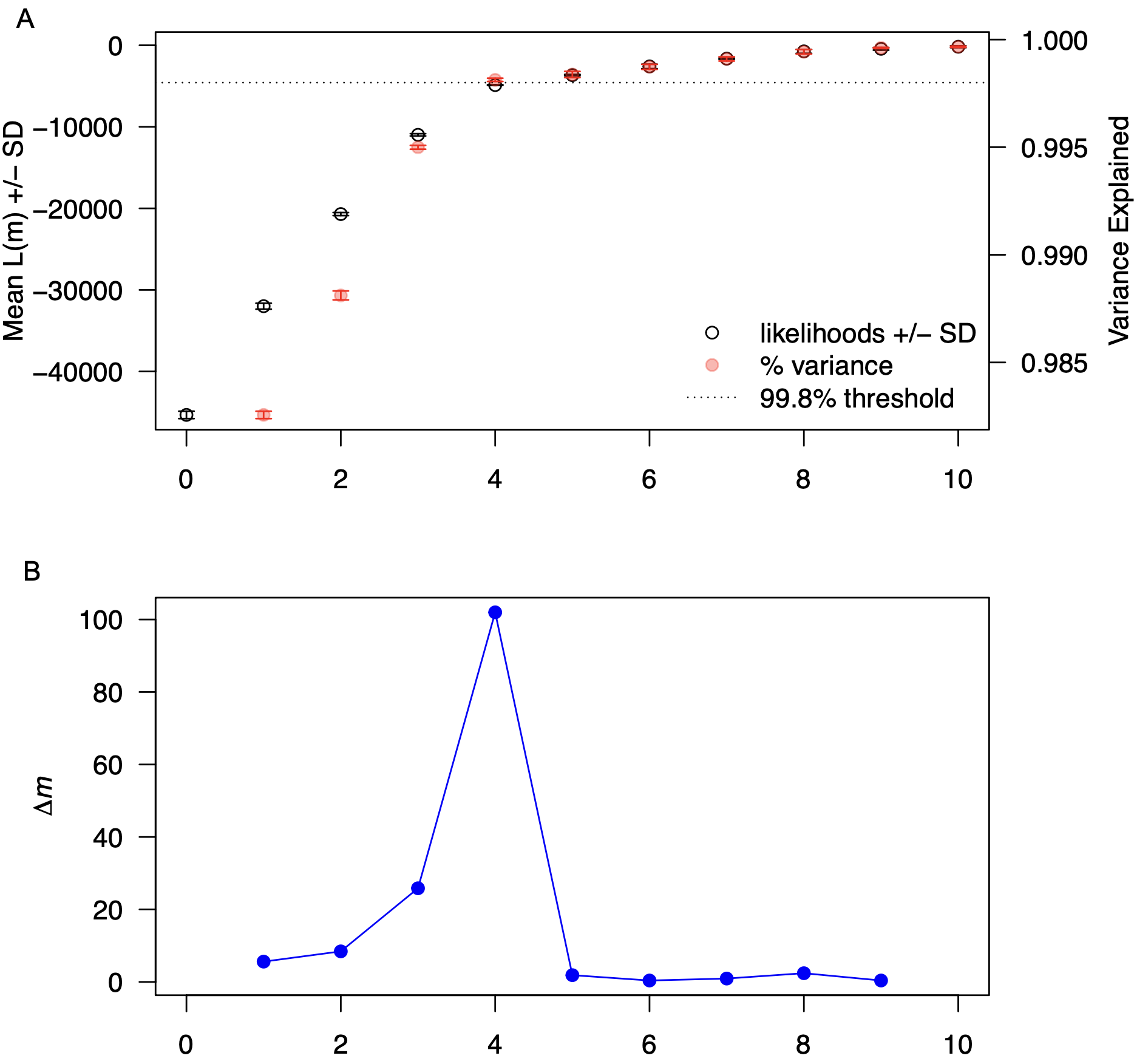


Figure S19.

**The results produced by OptM.** (A) The mean and standard deviation (SD) across 10 iterations for the composite likelihood L(m) (left axis, black circles) and proportion of variance explained (right axis, red circles). The 99.8% threshold (horizontal dotted line) is that recommended. (B) The second-order rate of change (∆m) across values of m. So m=4 was selected.


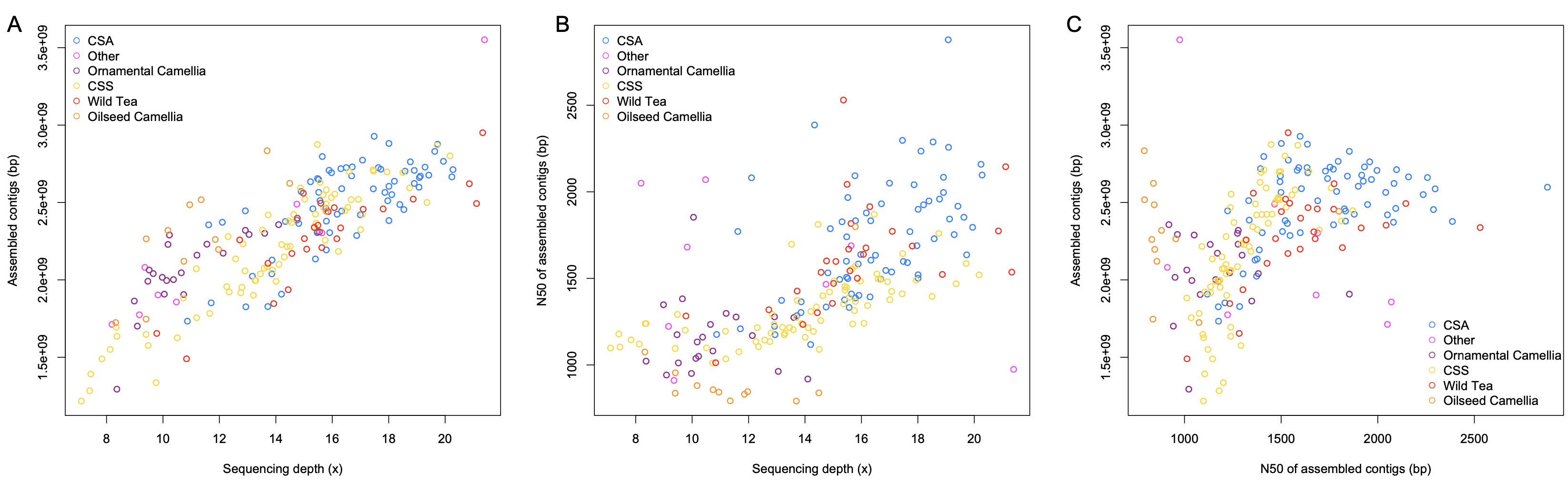


Figure S20.

**Summary of assembled results for pan-genome construction.** (A) Scatter plot of total sizes of assembled contigs and sequencing depths. (B) Scatter plot of N50 lengths of assembled contigs and sequencing depths. (C) Scatter plot of total sizes and N50 lengths of assembled contigs.


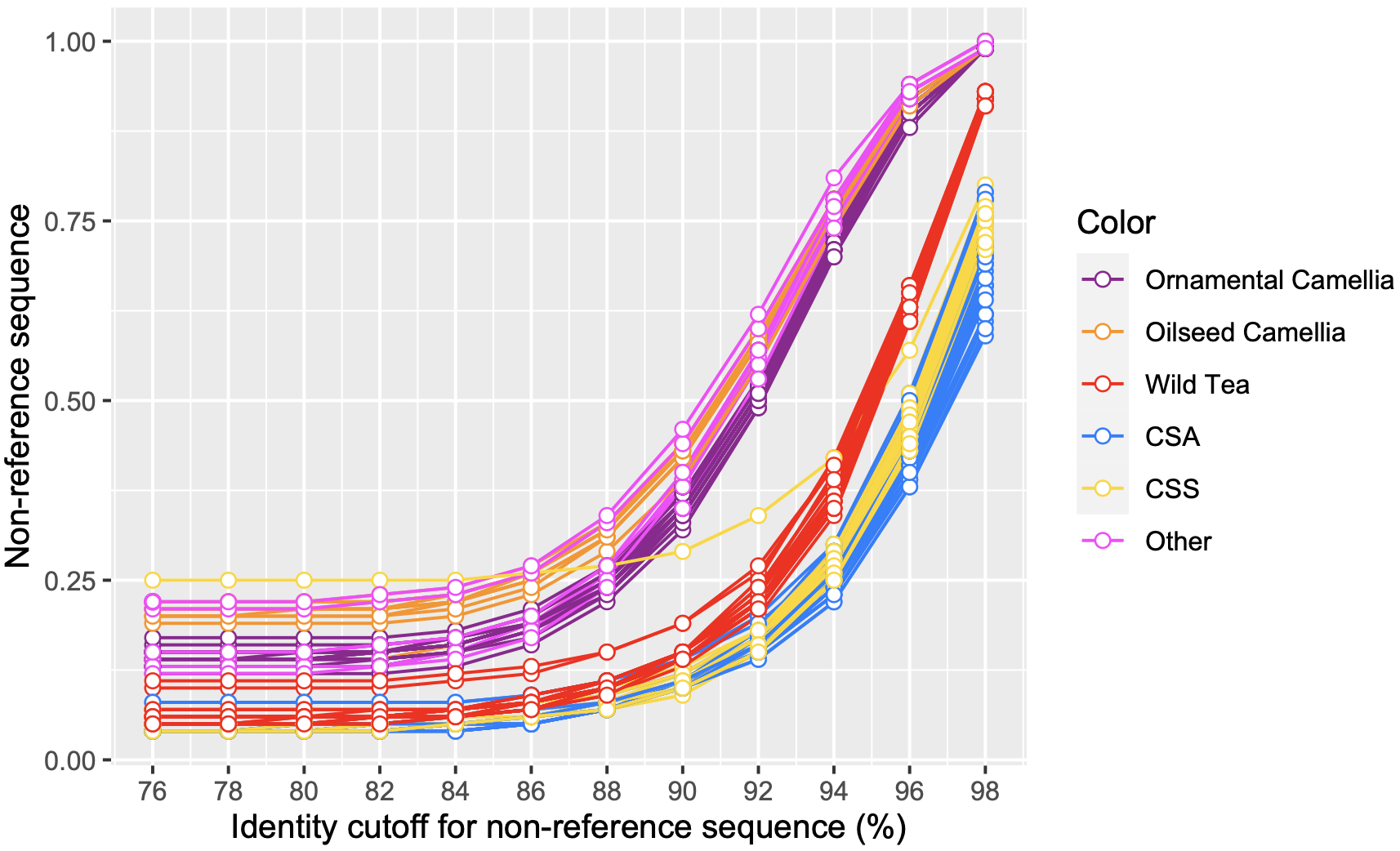


Figure S21.

**Variations for the non-reference sequences with different sequence identity thresholds.**


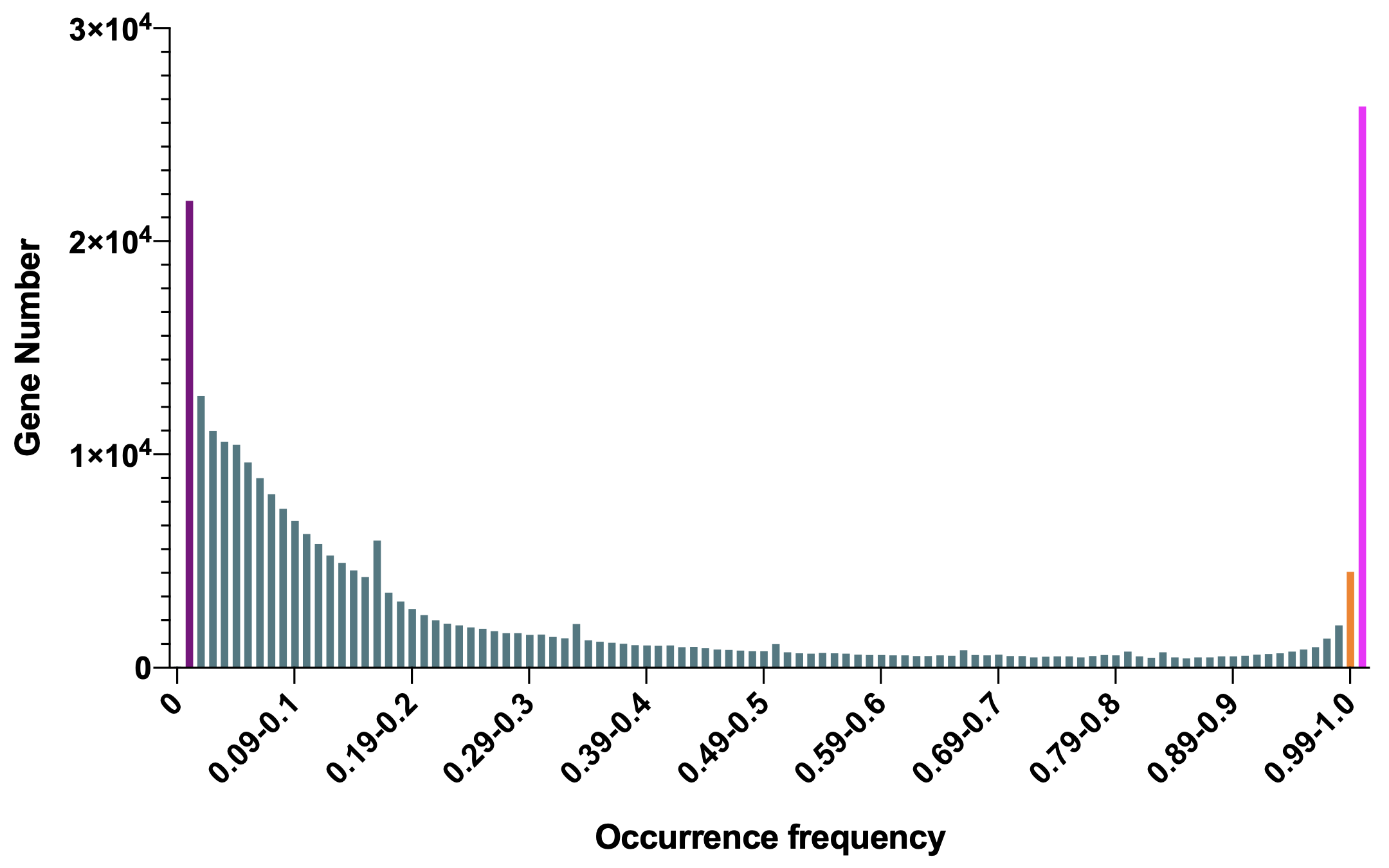


Figure S22.

**Composition of occurrence frequency for tea pan-genome.**


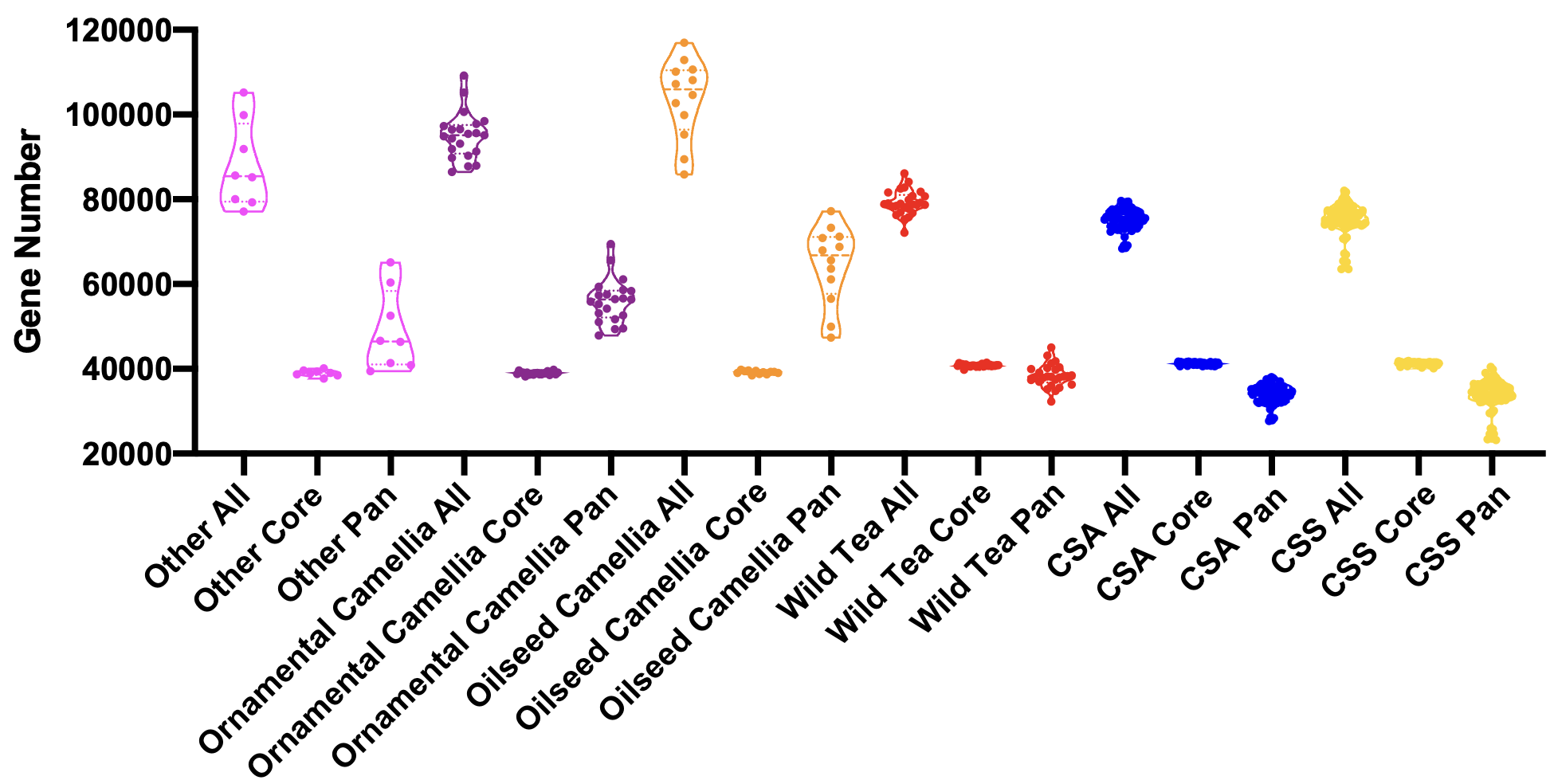


Figure S23.

**Violin plots showing the number of detected genes in each group.** Three lines (from the bottom to the top) in each violin plot show the location of the lower quartile, the median and the upper quartile, respectively.


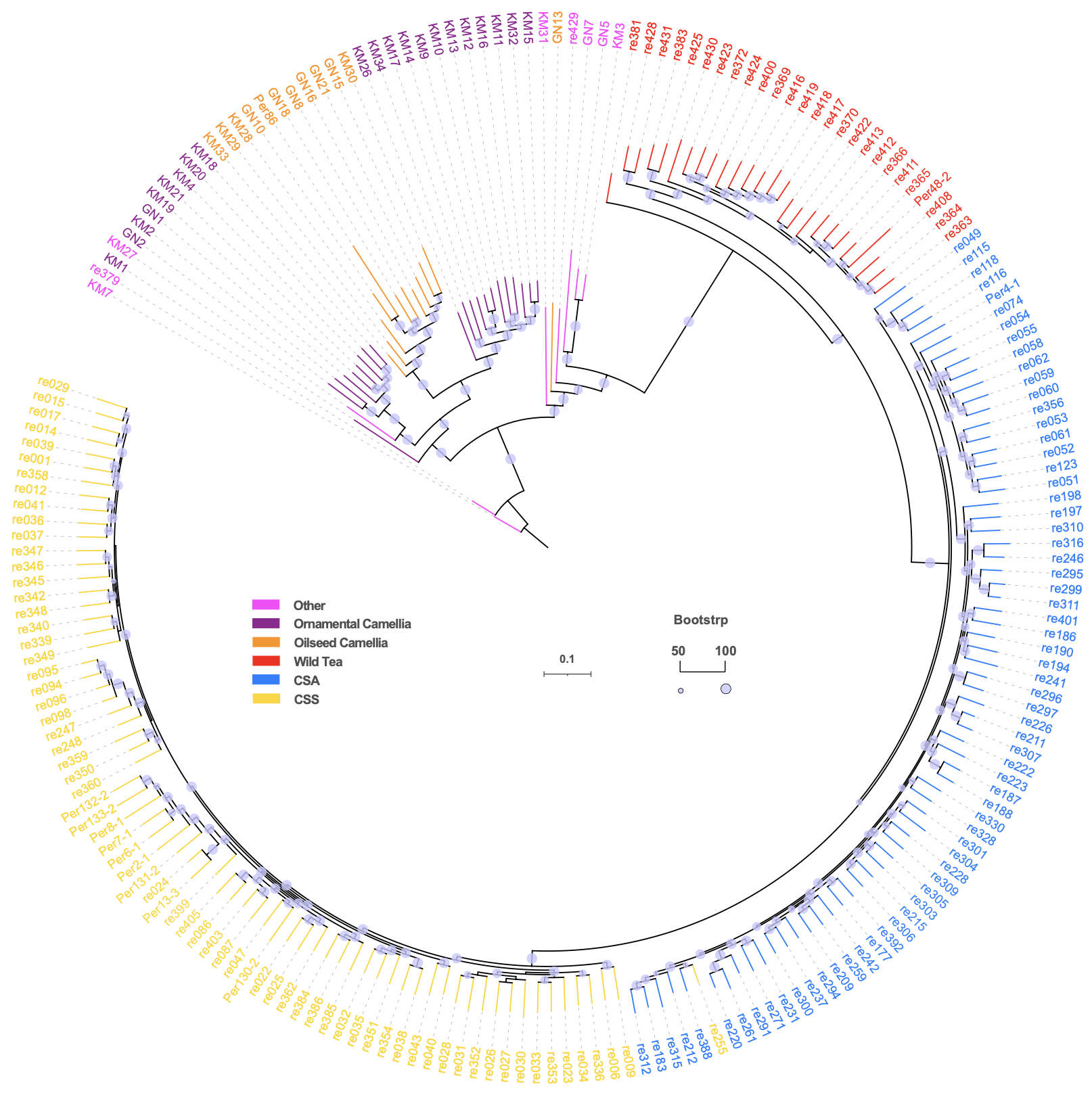


Figure S24.

**Phylogenetic tree of tea pan-genome.** The PAV data (0, absence; 1, presence) excluded all absent and core genes were used.


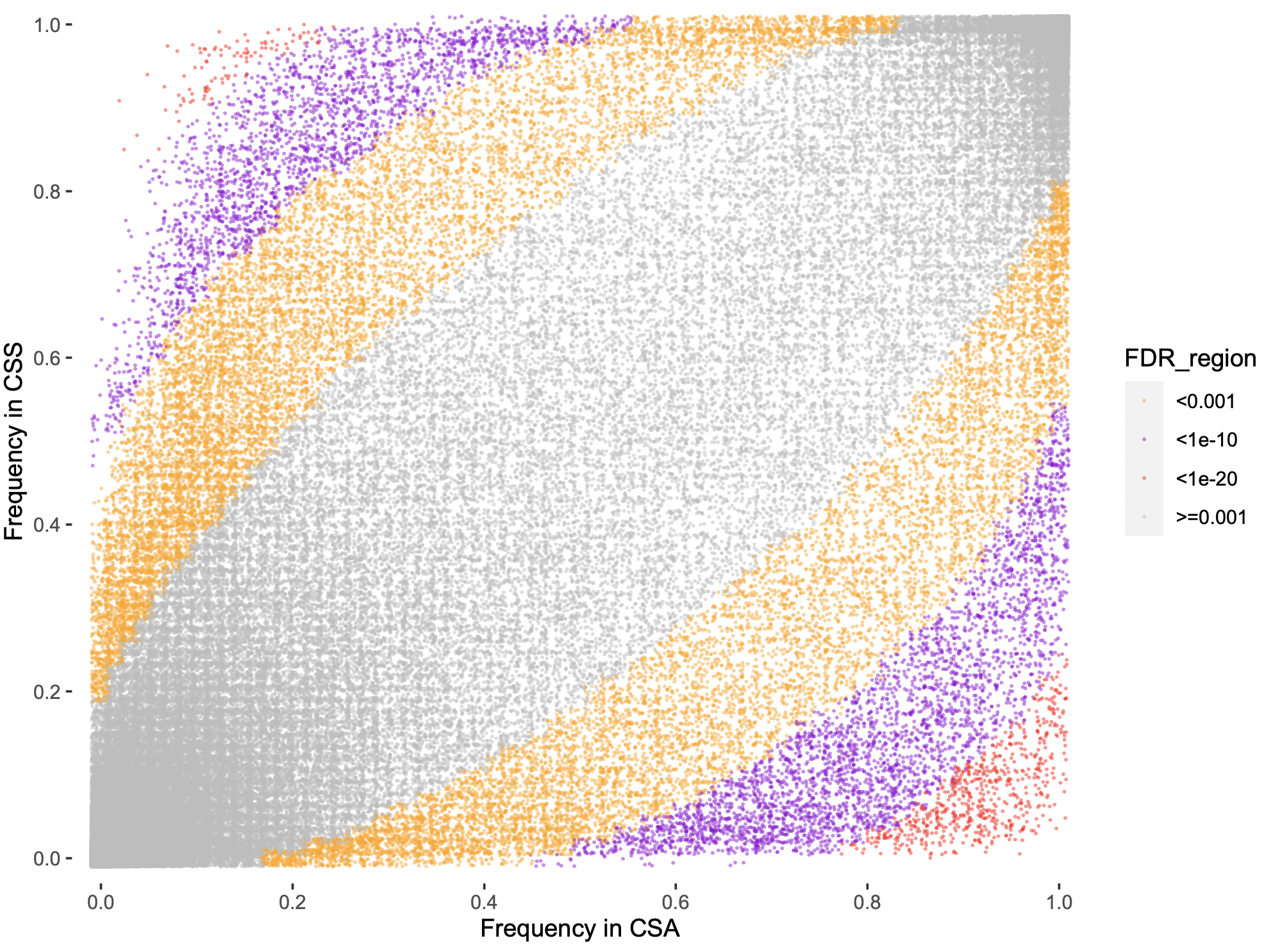


Figure S25.

**Scatter plots showing gene occurrence frequencies in CSA and CSS group.**


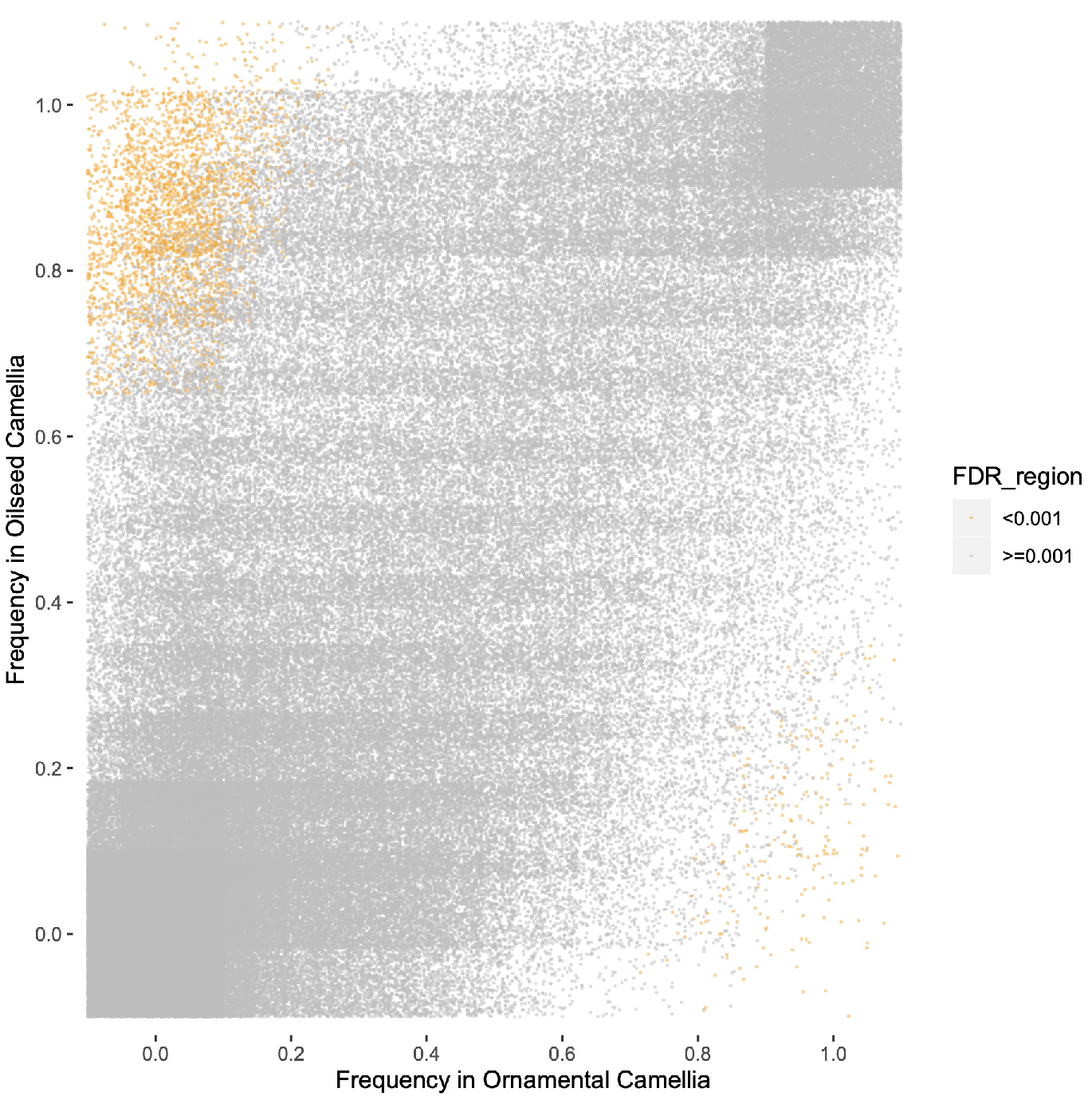


Figure S26.

**Scatter plots showing gene occurrence frequencies in ornamental *Camellia* and oilseed *Camellia* group.**
